# Oral vaccination stimulates neutrophil functionality and exerts protection in a *Mycobacterium avium* subsp. *paratuberculosis* infection model

**DOI:** 10.1038/s41541-021-00367-8

**Published:** 2021-08-12

**Authors:** Iraia Ladero-Auñon, Elena Molina, Maddi Oyanguren, Diego Barriales, Miguel Fuertes, Iker A. Sevilla, Lucy Luo, Rakel Arrazuria, Jeroen De Buck, Juan Anguita, Natalia Elguezabal

**Affiliations:** 1 Animal Health Department, Basque Institute for Agricultural Research and Development, NEIKER- Basque Research and Technology Alliance (BRTA), Derio, Bizkaia Spain; 2 Food Quality and Safety Department, Universidad del País Vasco/Euskal Herriko Unibertsitatea (UPV/EHU), Vitoria, Araba Spain; 3 Inflammation and Macrophage Plasticity Laboratory, CIC bioGUNE-Basque Research and Technology Alliance (BRTA), Derio, Bizkaia Spain; 4 Department of Production Animal Health, Faculty of Veterinary Medicine, University of Calgary, Calgary, AB Canada; 5 Ikerbasque, Basque Foundation for Science, Bilbao, Spain

**Keywords:** Inactivated vaccines, Innate immunity

## Abstract

*Mycobacterium avium* subsp*. paratuberculosis* (Map) causes paratuberculosis (PTB), a granulomatous enteritis in ruminants that exerts high economic impact on the dairy industry worldwide. Current vaccines have shown to be cost-effective against Map and in some cases confer beneficial non-specific effects against other pathogens suggesting the existence of trained immunity. Although Map infection is mainly transmitted by the fecal-oral route, oral vaccination has not been deeply studied. Therefore, the aim of this study was to compare the oral route with a set of mycobacterial and non-mycobacterial vaccines with a subcutaneously administered commercially available vaccine. Training effects on polymorphonuclear neutrophils (PMNs) and homologous and heterologous in vivo protection against Map were investigated in the rabbit infection model. Oral vaccination with inactivated or live vaccines was able to activate mucosal immunity as seen by elevation of serum IgA and the expression of *IL4* in peripheral blood mononuclear cells (PBMCs). In addition, peripheral PMN phagocytosis against Map was enhanced by vaccination and extracellular trap release against Map and non-related pathogens was modified by both, vaccination and Map-challenge, indicating trained immunity. Finally, PBMCs from vaccinated animals stimulated in vitro with Map antigens showed a rapid innate activation cytokine profile. In conclusion, our data show that oral vaccination against PTB can stimulate neutrophil activity and both innate and adaptive immune responses that correlate with protection.

## Introduction

Johne’s disease or paratuberculosis (PTB) is an infectious granulomatous enteritis that primarily affects domestic^1^ and wild^2^ ruminants, although it has been detected in other non-ruminant species including rabbits^3,4^ and birds, among others^5^.

*Mycobacterium avium* subspecies *paratuberculosis* (Map), the etiological agent of the disease, is an intracellular bacterium that is transmitted through the fecal-oral route. It ensures its survival and dissemination by infecting macrophages and exerting evasion mechanisms that permit its replication inside this immune cell. Macrophage infection triggers granuloma formation in the intestine, in particular in the ileum and ileocecal valve^6^.

The most common clinical signs of PTB are diarrhea, chronic weight loss, and decrease in production, which imply a premature elimination of animals and economic losses^7^. The control of this disease is based mainly on the improvement of herd management and the test and cull strategy, but vaccination has shown to be a cost-effective method^8^ against PTB, decreasing the number of clinical cases, reducing bacterial shedding in feces^9,10^ and improving milk production^10^. A commercial inactivated Map vaccine has shown heterologous protection against other mycobacterial infections^11^ and reduction in overall mortality in dairy cattle^12^, effects that could emerge from innate trained mechanisms or from cross-reactive T-cell mediated adaptive immunity. This vaccine has also shown priming of monocyte-derived macrophages as seen by increased Map killing activity compared to non-vaccinated animals^13^. All these findings suggest that trained immunity, such as that observed with the BCG vaccine in human newborns against *Mycobacterium tuberculosis* (Mtb), *Candida albicans*, and *Staphylococcus aureus*, can indeed be behind this effect^14^.

Despite the demonstrated beneficial effects of current commercial PTB vaccines, they are not allowed in cattle in most countries due to the interference with bovine tuberculosis (bTB) diagnostic tests^15^. Interference can be avoided or reduced by the introduction of variations in the diagnostic methods^15,16^, but this requires modifications in legislation that are difficult to achieve. Another approach to avoid interference with bTB diagnosis, is to apply vaccines through alternative routes as shown for an inactivated *M. bovis* (Mbv) vaccine in cattle^17^ and goats^18^ or a Map inactivated vaccine in guinea pigs^19^. In PTB, the oral route is a good alternative since it is the route of natural infection. However, mucosal immunity activation, disease protection, and absence of interference with bTB diagnosis through this delivery method need to be demonstrated.

Ruminant infection models of PTB are widely used for vaccination studies^20^. However, the lengthy incubation of the disease in ruminants added to the expenses of the experiments have led to the development of non-ruminant laboratory animal models. In this sense, rabbits which are naturally susceptible to infection with Map^3,4^ and have shown to be useful short term models to study the effect of diet on infection^21^ and to evaluate vaccination^22^, can be suitable to screen novel vaccines since many key aspects of PTB can be studied in this model^20^.

A better understanding of PTB pathogenicity and immune protection associated to vaccination is also needed in order to develop new effective vaccines. Several studies have addressed the importance of polymorphonuclear neutrophils (PMNs) in the innate immune protection against Mtb^23,24^ and very recently, BCG-induced protection has been shown to be dependent on PMNs^25^, showing they can undergo long-term functional reprogramming^26^. Additionally, the induction of specific Th1 and Th17 responses as a consequence of PMNs modulation by vaccination has demonstrated bacterial load reduction^27^, uncovering a “training capacity of PMNs”. The presence of PMNs at disease sites in the initial phases of mycobacterial infections has been demonstrated^28–30^. However, their role in defense against Map has only been suggested by recent transcriptomic studies describing the impairment of PMN recruitment and activation during PTB^31–33^. PMNs from healthy cattle have shown to exert extracellular trap (ET) formation and killing effects against Map in vitro^34^. The idea that proper PMN activation or training by PTB vaccines could eventually prevent or mitigate the effects of Map infection is a hypothesis that should be tested.

In this work we performed a vaccination-challenge Map experiment seeking evidence of protection against PTB via oral vaccination and possible trained immunity and cross- protection exerted by both mycobacterial and non-mycobacterial vaccines through ex vivo neutrophil functional assays. We have found that oral vaccination with inactivated or live Map vaccines is able to activate mucosal immunity as well as peripheral PMN functionality. In addition, oral vaccines showed increased neutrophil responsiveness to heterologous stimulations and an innate activation cytokine profile with protective effects against PTB in the rabbit Map infection model.

## Results

**Fig. 1:**
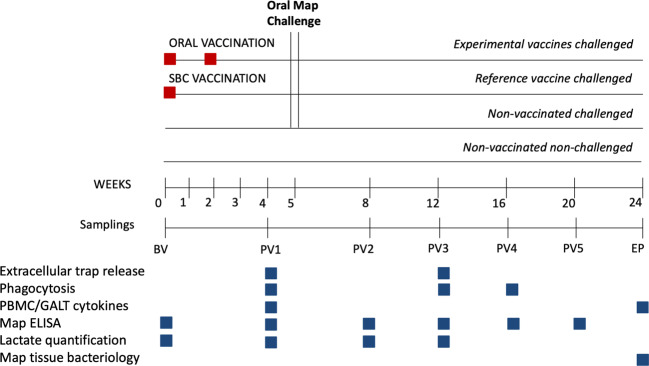
Experimental scheme. Interventions and sampling points used to study trained immunity in ex vivo functional assays and protection in vivo after infectious challenge with Map.

In order to assess mucosal and systemic immunity activation and disease protection through oral delivery we tested mycobacterial inactivated and live attenuated vaccines and a non-mycobacterial inactivated vaccine in the rabbit Map infection model following experimental scheme on Fig. 1. Specifically, an inactivated Map vaccine (MPV), a live attenuated vaccine (LAV), an *M. bovis* inactivated vaccine (MBV) and a *C. pseudotuberculosis* inactivated vaccine (CPV). In our experimental design, the isolation of PMNs from this combination of vaccinated animals along with homologous and heterologous stimuli would also permit assessing if these inactivated vaccines are capable of exerting training effects indicative of heterologous protection.

### Oral vaccination increases phagocytosis of Map by neutrophils

**Fig. 2:**
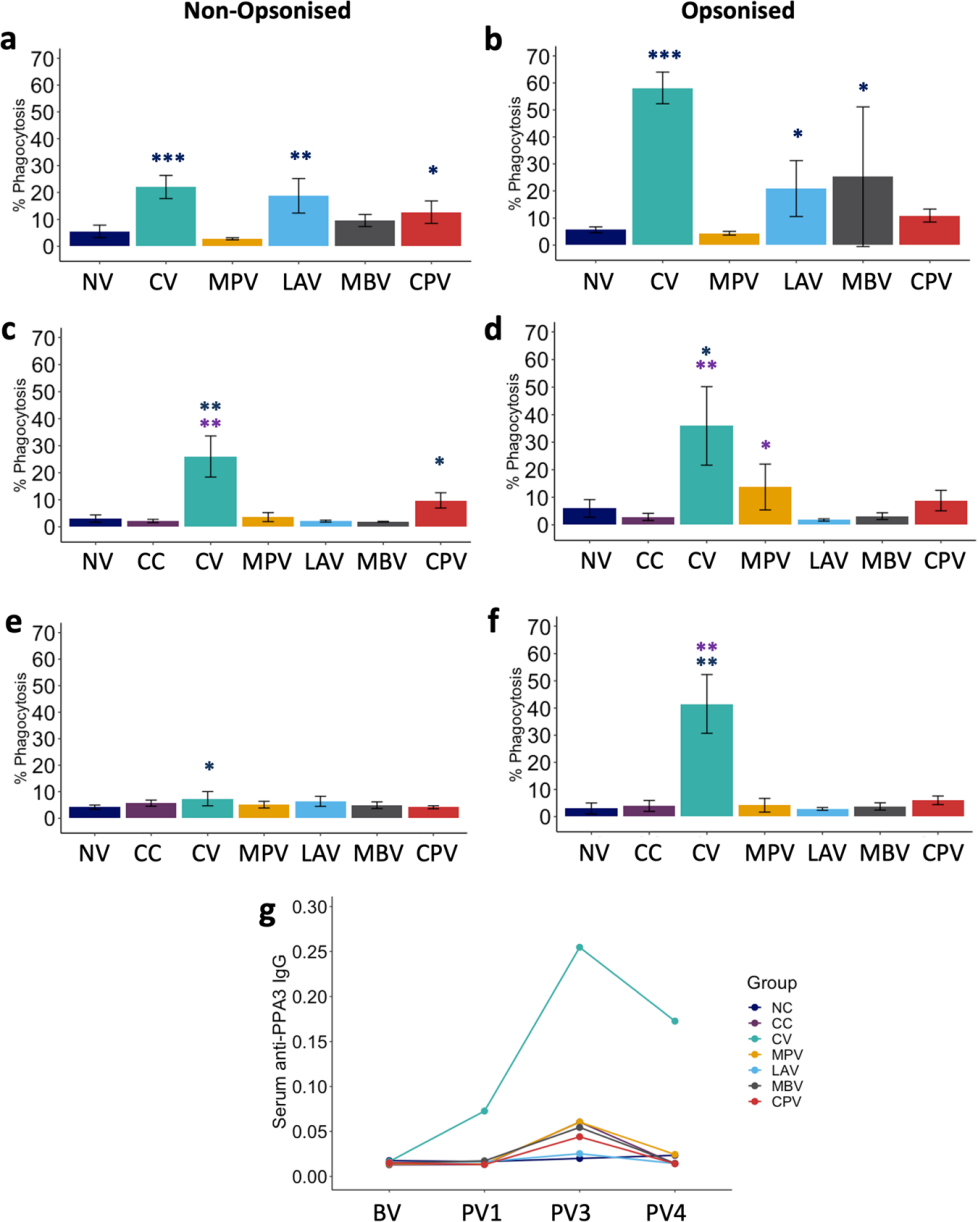
PMN phagocytosis levels. Phagocytosis against **a**, **c**, **e** non-opsonized and **b**, **d**, **f** opsonized *Mycobacterium avium* subsp. *paratuberculosis* (Map)-GFP at **a**, **b** PV1 (1 month after first dose vaccination), **c**, **d** PV3 (2 months after challenge) and **e**, **f** PV4 (3 months after challenge) of non-vaccinated and non-challenged (NV: NC+CC at BV and PV1, mean of *n* = 9); non-challenged (NC), non-vaccinated and challenged (CC), commercial Map inactivated vaccine (CV), killed Map strain oral vaccine (MPV), live attenuated vaccine (LAV), killed *Mycobacterium bovis* vaccine (MBV) and killed *Corynebacterium pseudotuberculosis* vaccine (CPV). **g** Serum anti-PPA3 IgG levels. All values for vaccinated groups were means of *n* = 5 with error bars representing standard deviation and Kruskal–Wallis with Dunn’s post-hoc test was applied. Blue asterisks represent significant differences compared to the NC group, purple asterisks compared to the CC group and signification levels are *<0.05, **<0.01, ***<0.001.

One of the main mechanisms that contribute to bacterial clearance by PMNs is phagocytosis so we assessed if oral vaccination would affect PMN phagocytic capacity. PMNs were isolated at different time points and exposed to Map-GFP ex vivo. Oral LAV and CPV and subcutaneous CV vaccination showed enhanced neutrophil phagocytosis against non-opsonized Map-GFP 4 weeks after first dose of vaccine administration (PV1) compared to non-vaccinated animals (NV) (Fig. 2a). This activity was maintained after Map challenge in some cases. At PV3 (12 weeks later) for CPV and CV vaccinated animals (Fig. 2c) and even after 16 weeks (PV4), a slight effect was still conserved for CV (Fig. 2e). Interestingly, CPV, a non-mycobacterial vaccine was able to activate PMNs for Map phagocytosis indicating that heterologous activation is possible.

Phagocytosis against opsonized Map-GFP with autologous plasma was increased in PMNs isolated from CV, LAV, and MBV vaccinated animals 4 weeks after the first dose at PV1 (Fig. 2b). PMNS from CV and MPV animals showed enhanced phagocytosis after Map challenge at PV3 (Fig. 2d), which was only maintained by CV PMNs at PV4 (Fig. 2f).

Map-GFP phagocytosis by PMNs correlated positively with anti-PPA3-antibody levels under opsonized conditions, as expected, at all sampling points (PV1: *ρ* = 0.77, *p* < 0.0001; PV3: *ρ* = 0.83 *p* < 0.0001 and PV4: *ρ* = 0.76, *p* < 0.0001). CV was the group with the highest phagocytic activity and Map-antibody titres. Oral vaccination was less efficient at maintaining a long-lasting elevated specific phagocytic activity of circulating PMNs and at boosting a specific humoral response (IgG and IgM) (Fig. 2g).

### Vaccination and Map challenge induce extracellular trap release by neutrophils

**Fig. 3:**
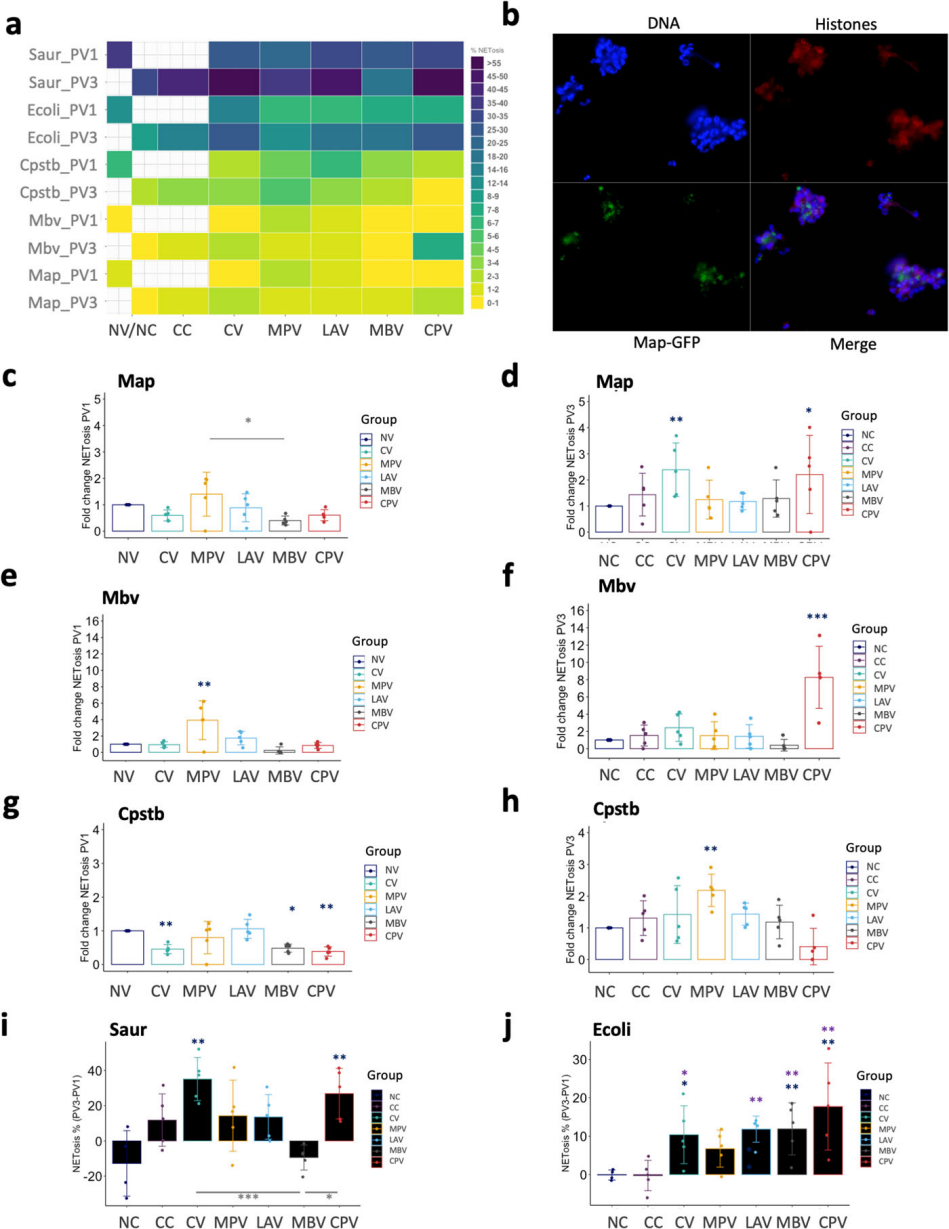
Neutrophil extracellular trap (NET) release levels. NET release against *Mycobacterium avium* subsp. *paratuberculosis* (Map), *Mycobacterium bovis* (Mbv), *Escherichia coli* (Ecoli), Staphylococcus aureus (Saur), and *Corynebacterium pseudotuberculosis* (Cpstb) of animals vaccinated with commercial Map vaccine (CV), killed Map oral vaccine (MPV), live attenuated vaccine (LAV), killed Mbv vaccine (MBV), and killed Cpstb vaccine (CPV). **a** Heatmap representation of raw % NETosis data. **b** Micrograph image (×40) showing ET release against Map-GFP. Fold changes referred to non-vaccinated (NV) animals against **c** Map, **e** Mbv and **g** Cpstb (at time-point PV1 (1 month after first dose vaccination) and against **d** Map, **f** Mbv, and **h** Cpstb referred to non-challenged (NC) animals at time-point PV3 (2 months after challenge). **i** ET release increase against Saur at PV3 compared to PV1 for all animal groups. **j** ET release increase against Ecoli at PV3 compared to PV1 for all animal groups. All values were means with error bars representing standard deviation from groups of *n* = 5, except for NC which was *n* = 4. ANOVA with Tukey’s post-hoc test was applied. Blue asterisks represent significant differences compared to the NC group, purple asterisks compared to the CC group and signification levels are *<0.05, **<0.01, ***<0.001.

PMNs from healthy animals are also capable of releasing ETs against pathogens such as *S. aureus*^35^, *E. coli*^36^, and *Map*^34^ or *Mbv*^34^, so we assessed if ex vivo NET formation was affected by vaccination and challenge against mycobacterial and non-mycobacterial microorganisms and NETosis levels were represented on a heat-map (Fig. 3a). PMNs isolated from animals vaccinated with MPV showed enhanced NET release against Map and Mbv (heterologous effect) at PV1 compared to NV (Fig. 3c, e). At this same time point all other tested vaccines showed decreased NET release compared to NV, suggesting that this mechanism is not predominant at this time point in those groups. At PV3 (two months after challenge and 3 months after first dose vaccination), NET release was increased in PMNs isolated from CV against Map (Fig. 3d), from CPV animals against Map and Mbv (again heterologous training effect) (Fig. 3d, f) and from MPV animals against Cpstb (also heterologous) (Fig. 3h). The combination of vaccination and challenge is probably what triggers an enhanced response of PMNs against Map in the CPV group.

Interestingly, PMNs from MBV (Fig. 3e, f) and CPV (Fig. 3g, h) animals showed an inhibition on ET release when stimulated ex vivo with the microorganisms that the vaccines were targeted for (secondary exposure), Mbv and Cpstb, respectively. This inhibitory effect was observed in both time-points PV1 and PV3 meaning that the challenge with Map did not affect PMN behavior of MBV and CPV against Mbv and Cpstb, respectively.

ET release against *S. aureus* (Fig. 3i) and *E. coli* (Fig. 3h) was generally increased at PV3 (2 months after Map challenge and 3 months after first dose vaccination) in all challenged groups.

ET release against Map and Mbv positively correlated both in PV1 and PV3, (PV1: *ρ* = 0.8, *p* < 0.001 and PV3: *ρ* = 0.57, *p* < 0.0001), although at PV3 correlation strength dropped, suggesting that although vaccination could exert innate cross protective effect in PMNs, in vivo challenge with Map could have reverted this effects or further changed some group’s PMNs behavior against mycobacteria.

### Vaccination enhances PMN aggregation trapping and surrounding Map in vitro

**Fig. 4:**
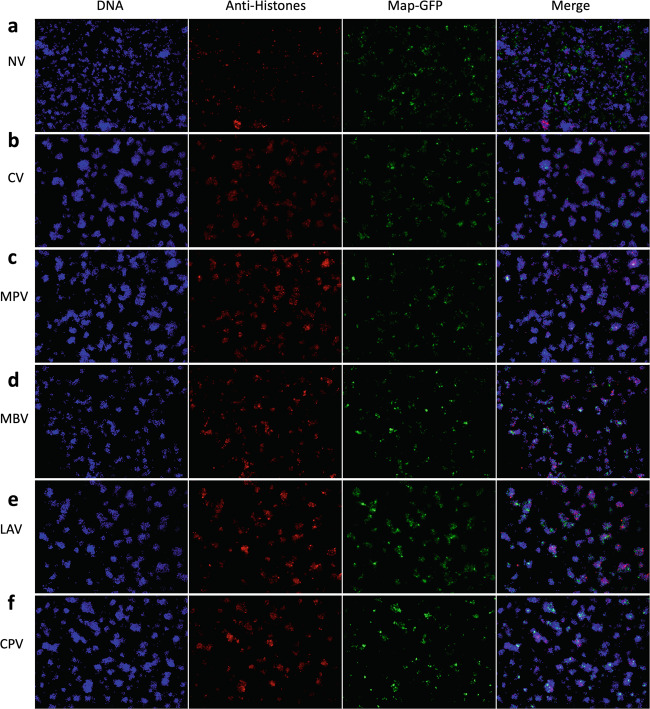
Micrographs of PMNs in contact with Map. **a** NV, **b** CV, **c** MPV, **d** MBV, **e** LAV, and **f** CPV at PV1 after 4 h incubation with Map-GFP (×10). DAPI (blue), anti-histone (red), and Map-GFP (green).

NETosis was also observed by microscopy (Fig. 3b). PMN behavior among experimental groups from PV1 varied after in vitro incubation for 4 h as seen in micrographs with Map-GFP (Fig. 4) and Map (Supplementary Fig. 5). PMNs from vaccinated groups aggregated around the mycobacteria, arranging to form bigger and more compact clusters compared to the NV group. The observed aggregates contained Map-GFP bacteria forming clumps surrounded by PMNs and skeins of chromatin threads. In the NV group, Map-GFP presence outside of PMN aggregates resulted more frequent and the observed clusters of PMNs were smaller and less compact.

### Vaccination elevates lactate levels in plasma indicating immune activation

**Fig. 5:**
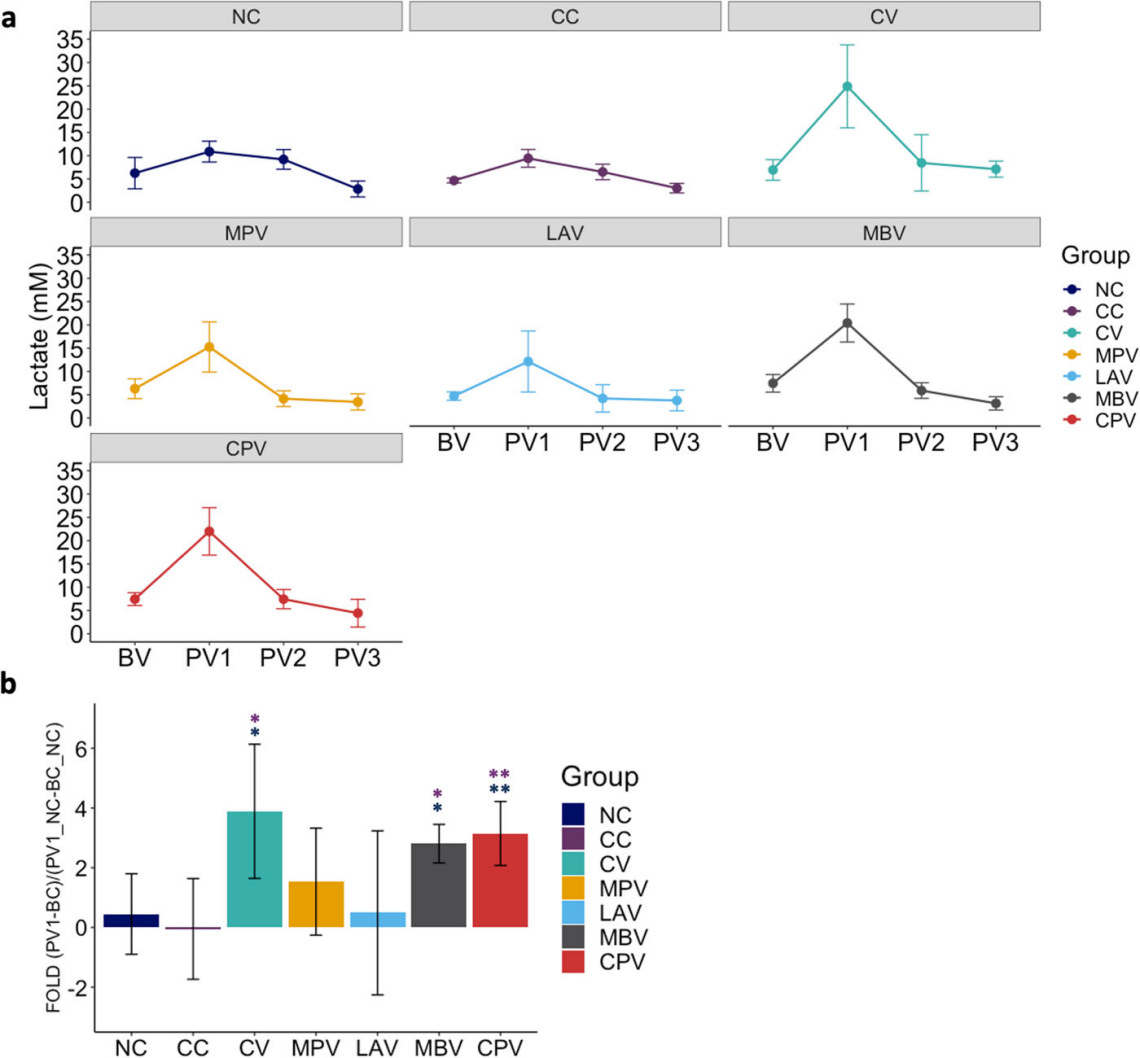
Lactate levels in plasma. **a** Lactate level changes in plasma throughout sampling points. **b** Lactate level increase of vaccinated groups with respect to NC during the period between PV1 (1 month after first dose vaccination) and BV (before vaccination). Blue asterisks represent significant differences between vaccinated or challenged groups with NC and purple asterisks represent significant differences between vaccinated groups and CC. All values were means with error bars representing standard deviation from groups of *n* = 5, except for NC which was *n* = 4. Kruskal–Wallis with Dunn’s post-hoc test was applied and signification levels are *p < 0.05, **p < 0.01.

There is evidence on the link between aerobic glycolysis and activated or proliferating cells, due to the increase in their metabolic demand^37^. In order to have more evidence on immune activation by oral vaccination, lactate levels were measured in peripheral plasma samples from BV, PV1, PV2, and PV3 samplings (Fig. 5a).

The increase in lactate at PV1 compared to BV was higher compared to the NC group for CV (*p* = 0.011), MBV (*p* = 0.019) and CPV (*p* = 0.009) (Fig. 5b) indicating a metabolic demand was taking place after vaccination. MPV also showed a considerable increase at PV1, though it was not significant (*p* = 0.161). On the contrary, LAV, a live Map vaccine did not show a significant increase of peripheral lactate levels. Afterward at PV2 and PV3 lactate dropped to baseline levels in all orally vaccinated groups. Lactate levels at PV3 were correlated with non-opsonized phagocytosis rates (*ρ* = 0.67, *p* < 0.00001).

### Cytokine expression profile and IgA levels suggest that oral vaccines activated mucosal immunity

**Fig. 6:**
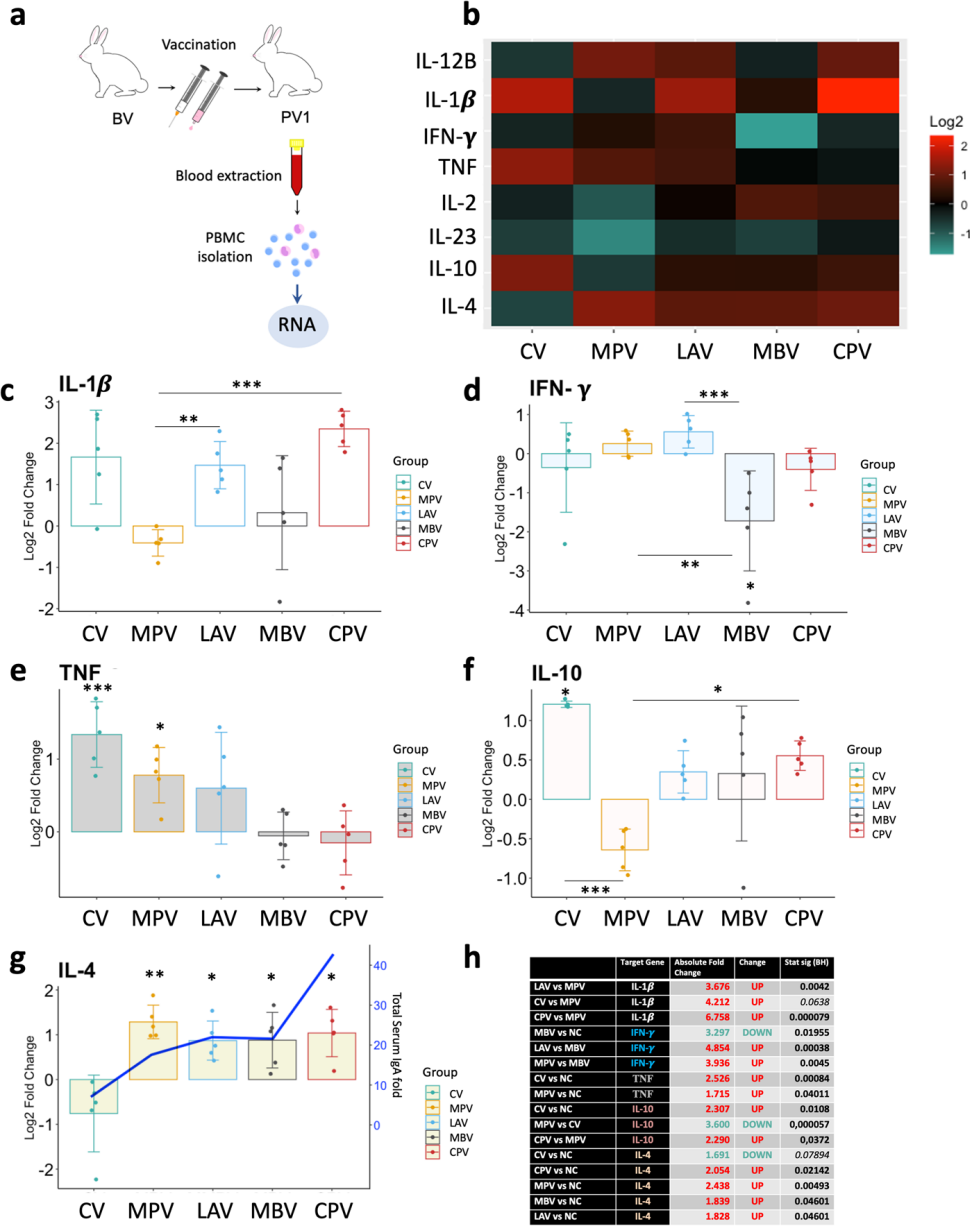
Cytokine relative quantification by RT-qPCR of PBMCs isolated from animals at PV1 (1 month after first dose vaccination) relative to the NC. **a** Schematic representation of sample origin. **b** Heatmap representation of cytokine expression of vaccinated groups; in red upregulated and in green downregulated gene expression. Bar charts showing log2 fold change between vaccinated groups and NV. **c** IL-1β, **d** IFN-γ, **e** TNF, **f** IL-10, **g** IL-4 and blue line represents serum total IgA level fold PV1-BV. **h** Table summarizing the significant differences between groups in cytokine expression and the Absolute Fold Change Factor. All values were means with error bars representing standard deviation from groups of *n* = 5, except for NV which was *n* = 5. ANOVA with pooled SD with Benjamini & Hochberg correction was applied and signification levels are *p < 0.05, **p < 0.01, ***<0.001.

In order to characterize the cytokine expression in PBMCs stimulated by vaccination, gene expression levels of IFN-γ, TNF, IL-2, IL1-β, IL- 4, IL-12B, IL-10, and IL-23A cytokines were assessed in isolated PBMCs at 1 month after first vaccination dose (PV1) (Fig. 6a) and the response of the vaccinated groups were quantified relative to the NC group, and represented on a heat-map (Fig. 6b) and further analyzed.

Orally administered LAV, is an attenuated vaccine and the only live vaccine included in the study. PBMCs from this group presented high levels of IL-1β (Fig. 6c) and the highest basal expression of IFN-γ at PV1 (Fig. 6d) indicating a Th1 response. This is partially expected since it shows the nearest infection-like profile compared to the rest of the groups. On the other hand, CV subcutaneous vaccination presented the highest basal expression of TNF (Fig. 6e) and IL-10 (Fig. 6f). In contrast, MPV was the group with the lowest IL-10 expression.

As early as 1 month after the first vaccination dose, all oral vaccines enhanced IL-4 basal expression compared to NC (Fig. 6g). MPV was the group with the highest IL-4 basal expression, followed by CPV, MBV, and LAV. In contrast, the subcutaneously administered CV group presented the lowest basal expression of IL-4.

This IL-4 elevation suggests that oral vaccines from our study had activated mucosal immunity. IL-4 elevation is necessary for mucosal and systemic immunity activation and IgA production^38^. Serum IgA levels have been used to predict both vaccine efficacy and the immune status of individuals or herds against enteric infectious diseases^39^, so we decided to measure total IgA in serum at BV and PV1 hypothesizing that oral vaccination would have activated the production of this antibody isotype. Indeed, serum IgA levels increased only in orally vaccinated animals (Fig. 6g, blue line), showing highest increase for CPV.

### MPV vaccine shows the most convenient basal profile of immune modulation whereas CPV induced highest levels of IL-1β

Taking into account the expression of the studied cytokines, MPV is the group showing the best basal pro-inflammatory/Th2 balance at PV1, showing the highest IL-4 expression, a significant TNF increase, and the lowest IL-10 expression. The group showing the highest basal levels of IL-1β was CPV, in contrast to MPV, which showed the lowest levels, while IL-10 expression was moderate compared to the rest of the groups.

### IL-1β basal increase correlated with increased phagocytosis activity of neutrophils

IL-1β basal levels and phagocytosis at PV1 were positively correlated (non-opsonized: spearman *ρ* = 0.54, *p* = 0.002; opsonized Map: spearman *ρ* = 0.57, *p* = 0.001). This is partially expected since PBMC derived IL-1β plays a role in neutrophil recruitment, activation and inhibition of neutrophil apoptosis^40^.

### Exposure of PMBCs to Map antigens in vaccinated groups induces an innate immune activation profile

**Fig. 7:**
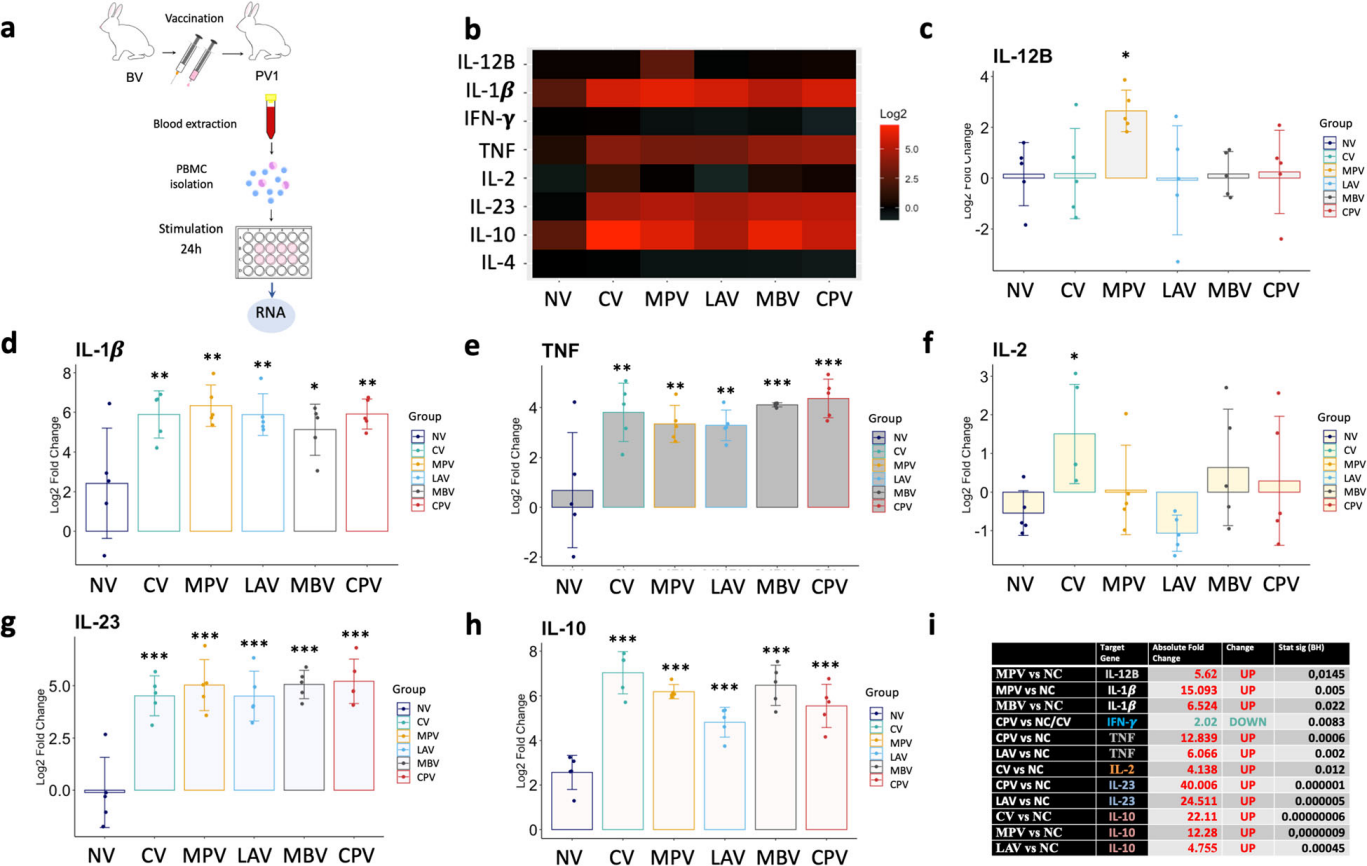
Cytokine relative quantification by RT-qPCR of PBMCs isolated from animals at PV1, after ex vivo stimulation with Map sonicate (SM), of the studied vaccination groups relative to their correspondent non-stimulated control (PBS). **a** Schematic representation of sample origin. **b** Heatmap representation of the average expression of cytokines; in red upregulated and in green downregulated expression. Bar charts showing log2 fold change between groups. **c** IL-12B, **d** IL-1β, **e** TNF, **f** IL-2, **g** IL-23A, **h** IL-10. **i** Table summarizing significant differences between groups in cytokine expression and the Absolute Fold Change Factor. For IL-1β, TNF, IL-23A, and IL-10 only the higher and the lower Absolute Fold Change and *p*-values are detailed. All values were means with error bars representing standard deviation from groups of *n* = 5, except for NC which was *n* = 4. ANOVA with pooled SD with Benjamini & Hochberg correction was applied and signification levels are *p < 0.05, **p < 0.01, ***< 0.001.

In order to assess the impact of vaccination on the cytokine expression in PBMCs after ex vivo exposure to Map antigens, gene expression was relatively quantified from PBMCs isolated at PV1 and stimulated with Map sonicate (SM) for 24 h with respect to the expression without stimulation (PBS) (Fig. 7a) and represented on a heat-map (Fig. 7b).

All the vaccinated groups showed an innate immune activation response (elevated IL-1β, TNF, IL-23A) after exposure to Map antigens compared to the NC group (Fig. 7d, e, g). IL-10 was also highly increased in all vaccinated groups indicating regulation of the inflammatory response (Fig. 7h). Actually, no increase of IFN-γ, nor IL-4 expression was achieved in any group with respect to their non-stimulated control. These results can be explained by the high expression of IL-23, which promotes the differentiation of naive CD4+ T cells into Th17 cells that produce IL-6, IL-17, IL-17F, and TNF, but not IL-4 and IFN-γ^41^.

On the other hand, MPV showed the highest IL-12B levels (Fig. 7c) and CV showed the highest IL-2 values (Fig. 7f).

### A discrete pro-inflammatory polarization was observed in GALT from challenged animals and CV showed the most lymphoproliferative profile

**Fig. 8:**
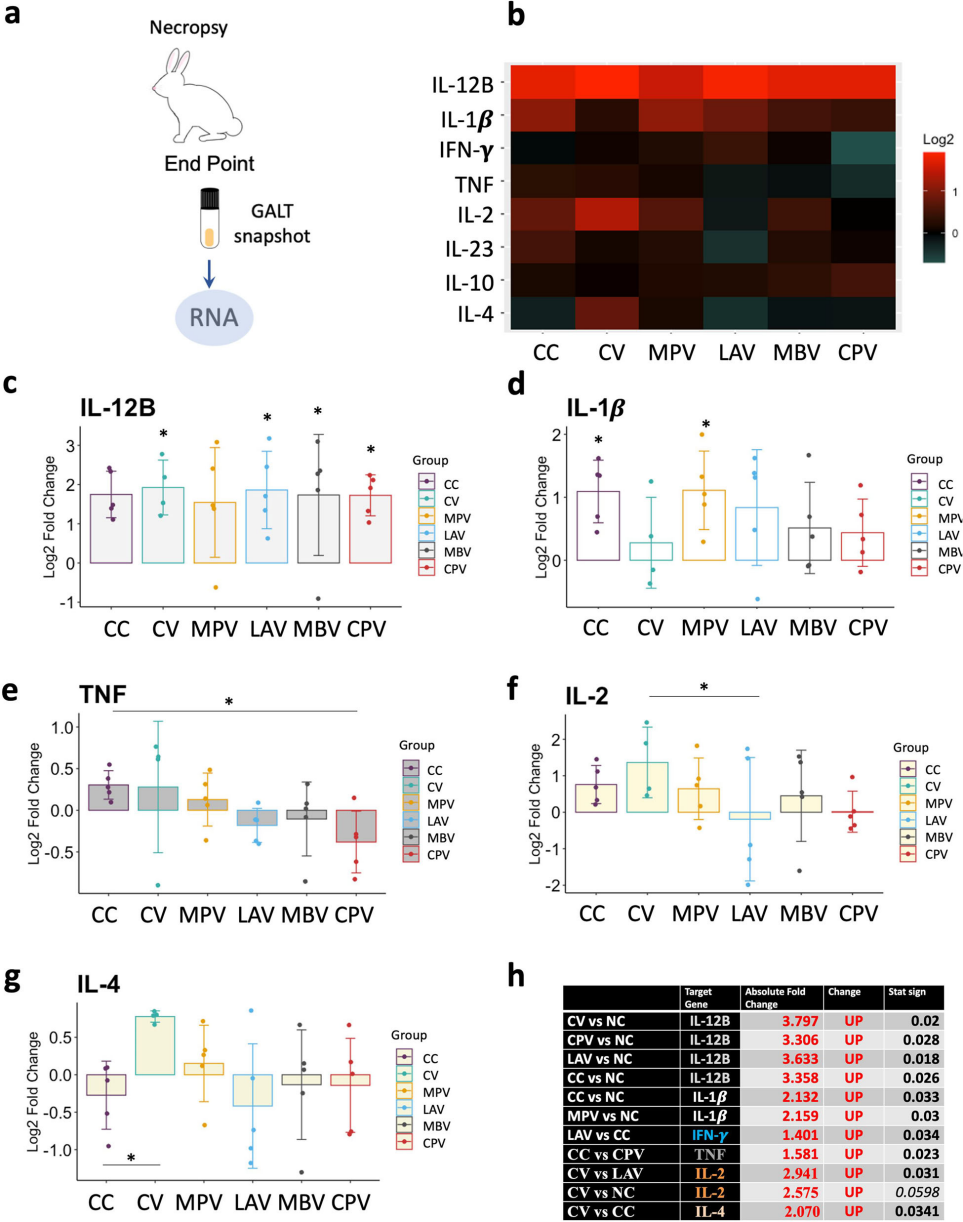
Cytokine relative quantification by RT-qPCR in GALT at the end point of the experiment of all challenged groups relative to the NC. **a** Schematic representation of sample origin. **b** Heatmap representation of the average expression of cytokines; in red upregulated and in green downregulated gene expression. Bar charts showing log2 fold change between groups. **c** IL-12B, **d** IL-1β, **e** TNF, **f** IL-2, **g** IL-4, h IL-10, **i** Table summarizing significant differences between groups in cytokine expression and the Absolute Fold Change Factor. All values were means with error bars representing standard deviation from groups of *n* = 5, except for NC which was *n* = 4. ANOVA with Tukey’s post-host test was applied and signification levels are *p < 0.05, **p < 0.01, ***<0.001.

In order to profile local immune response to Map at the final stage of the experiment expression of the same cytokines studied in the peripheral response was analyzed from GALT (Fig. 8a) and challenged groups’ responses were relatively quantified with respect to the NC group and represented on a heat-map (Fig. 8b).

In general, all challenged groups showed a more pro-inflammatory profile in the GALT at the end-point compared to the NC group, based on the higher local expression of IL-12B in these groups (Fig. 8c). IL-1β expression was also slightly higher for all challenged groups compared to the NC except for CV (not oral vaccine) (Fig. 8d). However, only CC and MPV showed significant differences.

IL-2 considered the major T lymphocyte growth factor, also necessary for memory lymphocyte T differentiation^42^. In this study, the highest local expression of IL-2, was shown by the CV group (Fig. 8f). This increase in IL-2 levels could be an indicator of vaccine efficacy since it plays an important role in the initiation and maintenance of antigen-specific immune responses^43^. CV also showed higher local IL-4 expression compared to CC, indicative of lymphocyte B proliferation (Fig. 8g).

The expression of IL-12B and IL-2 resulted positively correlated (*ρ* = 0.42, *p* < 0.0001) and the strength of the correlation between IL-12B and IL-2 increased in GALT of those animals with demonstrated Map colonization of tissues (*ρ* = 0.73, *p* = 0.003).

### MPV and CPV vaccinated animals presented lowest Map tissue colonization

In order to partially assess protective effects of the oral vaccines in this subclinical model of infection, Map isolation from tissues was performed on two different culture media augmenting the isolation probability and data are shown on Tables 1 and 2.

**Table 1:** Bacteriological analysis results of the control groups

Group	NC				CV					CC				
ID	31	33	34	35	1	2	3	4	5	26	27	28	29	30
HEYM	–	–	–	–	–	–	–	–	SAC	–	SAC	–	VA	VA
TiKa	–	–	–	–	–	–	–	–	–	–	MLN	–	–	–

Negative results with “–“ symbol; positive results show with abreviations indicating the sample tissue: Sacculus rotundus (SAC), vermiform appendix(VA), mesenteric limph node (MLN).

**Table 2:** Bacteriological results of oral vaccination groups

Group	MPV					LAV					MBV					CPV				
ID	11	12	13	14	15	21	22	23	24	25	16	17	18	19	20	6	7	8	9	10
HEYM	–	–	–	–	–	SAC	SAC, VA	–	–	–	–	VA	SAC	SAC	SAC	–	SAC	VA	–	–
TiKa	–	–	MLN	–	–	SAC	–	–	SAC	–	–	MLN	–	–	MLN	–	–	–	–	–

Negative results with “–“ symbol; positive results show with abreviations indicating the sample tissue: Sacculus rotundus (SAC), vermiform appendix(VA), mesenteric limph node (MLN).

Map was not isolated in any samples from the NC group as expected. Only one GALT tissue (SAC) from the CV tested positive in HEYM culture. In the CC group three out of five animals were positive for Map in HEYM or TiKa. The best results amongst orally vaccinated groups are those from MPV and CPV with only one and two positive tissues, respectively.

LAV positive cultures in HEYM or TiKa were re-cultured in 7H9 OADC MJ with hygromycin. No colonies were observed confirming that colonies from tissue cultures from this group belonged to the challenge strain.

### Principal component analysis of immune parameters shows a homogeneous and well-differentiated immune shaping by MPV and CPV oral vaccines

**Fig. 9:**
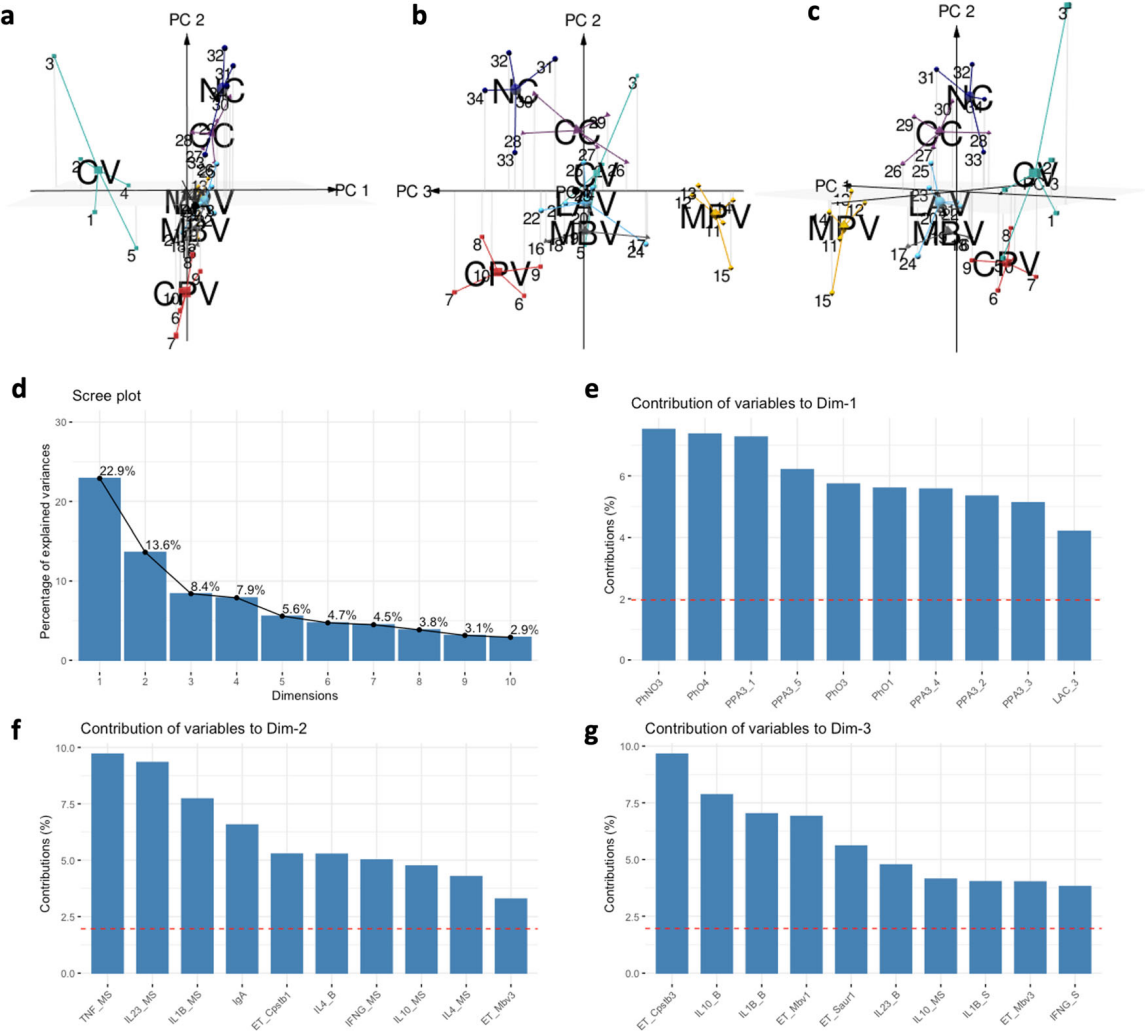
Principal component analysis maps. **a**–**c** 3D representation of the spatial disposition that individuals adopt defined by the set of immune variables assessed in the experiment. **d** Percentage of explained variances by the top 10 dimensions of the analysis. **e** Contribution of variables to Dimension 1; phagocytosis, anti-PPA3 IgG, and lactate levels explain 22.9% of the variation. **f** Contribution of variables to Dimension 2; cytokine expression of stimulated and non-stimulated PBMCs explain 13.6% of the variation. **g** Contribution of variables to Dimension 3. Cytokine expression levels of sacculus rotundus and lactate (PV1 and PV2) had no impact on the three main principal components of the analysis. All parameters were from groups of *n* = 5, except for NC which was *n* = 4.

In order to visualise the global impact of vaccination immunological parameters were considered for a principal component analysis (PCA). Although Map isolation from tissues was not considered for the PCA, the groups that the analysis located in the center (LAV, CC, and MBV) were coincident with the groups showing highest Map tissue positivity, while the groups located in the periphery were the groups with most Map negative tissues (NC, CV, CPV, and MPV). Moreover, CV, CPV, and MPV, were also represented separately from each other, reflecting the differences in their immune modulation outcome probably due to the differences in vaccine composition and administration route (Fig. 9a–c).

The CV vaccinated group displays a large and distant ellipse, consistent with the strong, but heterogeneous effects that are seen in the group (Fig. 9a). This vaccine triggers different activation levels in the animals, showing, for instance, high antibody titres in some animals and low values in others. In contrast, oral MPV and CPV, display more compact ellipses, indicating moderate and homogeneous effects in the groups (Fig. 9b).

CC and NC are closely displayed (Fig. 9b, c), probably because Map tissue culture has not been considered, Map replicates slowly inside macrophages intending to evade the immune system, thus, provoking minimum reaction is beneficial for its’ survival. Also, both groups undergo less stimulations in contrast to the vaccinated groups.

The impact of the parameters on the principal components is shown on Fig. 9d–g. In general, this analysis shows that oral MPV and CPV vaccines, led to homogeneous responses, and a singular response, far from NC and CC, comparable to the subcutaneously administered CV commercial vaccine, indicating that these can be promising candidates for oral vaccination.

## Discussion

PTB is a worldwide disease mainly affecting domestic ruminants’ health and production, but also posing a potential zoonotic threat^44^. Available commercial vaccines show good results in field studies^8^. Also, beneficial non-specific effects in terms of overall mortality reduction in vaccinated herds have been reported^12^, although the underlying immunological mechanisms behind these non-specific effects have been object of research only recently^13^. Besides, these vaccines present some negative aspects to overcome, such as, some interference with bTB diagnostic tests^15^. This problem may be solved by changing the vaccination route from subcutaneous to oral^19^, though mucosal immunity activation through this route must be tested. In our study we have shown that mucosal activation is possible through oral vaccination (even with inactivated vaccines), and that vaccination against PTB with experimental vaccines (Map inactivated and *C. pseudotuberculosis* inactivated) and with a Map commercial vaccine is able to enhance neutrophil functionality (showing trained immunity effects) as seen by increased phagocytosis and ET release, important mechanisms for bacterial clearance.

PMNs are able to efficiently phagocytose pathogens by opsonization with complement factors or antibodies^45^. Also, bovine PMNs have been reported to express several membrane TLRs^46^, which have shown to increase phagocytosis^47^. In our study, we observe an increased non-immune phagocytosis (in the absence of opsonins) by PMNs of orally vaccinated rabbits, which is most prominent one month after vaccination but temporary, lasting up to 3 months after vaccination for vaccine CPV. This non-opsonized phagocytosis could be linked to an increased expression of pattern recognition receptors (PRPs) on the surface of PMNs from vaccinated animals as seen in PMNs from BCG vaccinated humans that show anti-bacterial functions for at least 3 months after vaccination and changes in PMN phenotype, toward the expression of activation markers^26^. Also, lactate levels were correlated with non-opsonized phagocytosis in PV3, being the CV group the main contributor to this correlation. It may be that CV is inducing high levels of granulopoiesis in bone marrow that require elevated aerobic glycolysis resulting in lactate increase, phenomenon that has also been reported for BCG^48^.

Phagocytosis of antibody-opsonized bacteria was higher than non-opsonized phagocytosis in most cases and highest in the CV control group correlating with higher levels of specific anti-Map-antibody levels in plasma. In PTB, the possible protective effect of antibodies has been questioned and the shift to humoral response has been related to clinical stages of the disease and severe lesions^49^. However, recent studies have demonstrated that the humoral response is necessary for vaccine protection^50^. Our findings suggest that during the first months after vaccination with CV PMN functionality is increased, we hypothesize that activation may occur through upregulation of surface PRPs but once antibody levels increase, immune phagocytosis would be predominant and, in this case, antibodies would be helpful if phagocytes encounter extracellular Map.

ET formation by PMNs is an important effector mechanism for pathogen clearance^51^. ET release results presented in this study suggest a certain degree of immunity training exerted by oral vaccination that correlates with protection. Firstly, groups with the lowest Map tissue positivity (CV, CPV, and MPV) show, at some point (one month or three months after vaccination) higher ET release than the non-infected control group when challenged in vitro with Map. Secondly, PMNs from CPV and CV groups are the ones showing the highest ET release, which also happened to be the groups showing the best PMN phagocytosis response, these are the groups with highest PMN activation or training levels.

Interestingly, in some cases, this training or functional activation is heightened by Map oral challenge. One month after vaccination (PV1), PMNs of all orally vaccinated groups, showed lower ET level release against *E. coli* compared to CV and control groups, whereas after Map challenge (PV3) ET release levels increased for all challenged groups. This phenomenon was also observed against *S. aureus*. Also, worth mentioning is the finding that vaccines CPV and MBV did not show antigen-specific response to self antigen. This could be due to some type of self tolerance as that seen in macrophages when low doses of LPS fail to activate histone marks in promoters of genes involved in phagocytosis pathways resulting in a lower inflammatory response of macrophages to subsequent stimulation^52^ or the activation of different defense mechanisms due to vaccination.

What takes PMNs to release ETs or to phagocytose pathogens remains a mystery^53^ and many microorganisms have not been tested yet for their ability to stimulate ET release. Actually, this is the first time NETosis has been reported against Cpstb in vitro. Interestingly, high ET release is not always associated with the control of the disease^54^. Indeed evidence is accumulating that excessive production of NETs is related to the exacerbation of inflammation and the development of autoimmunity, cancer metastasis, and inappropriate thrombosis^55^. We believe that a good PTB vaccine should potentiate phagocytosis, rather than NETosis considering that Map is an intracellular bacterium.

Oral vaccines enhanced early IL-4 expression in circulating PBMCs, which is known to be a relevant cytokine for mucosal immunity shaping^38^. Added to this, total serum IgA increased after vaccination in the oral vaccinated groups pointing in the same direction being highest for CPV, which also shows high PMN activation and low Map tissue positivity. On the contrary, the subcutaneous administration of CV did not elevate either IL-4 or serum IgA. CV caused an increase in IL-10, and also high TNF and low IL-4 expression in circulating PBMCs we hypothesize due to granuloma formation at the inoculation site as seen for Mycopar®, another commercially available whole-cell bacterin containing inactivated Map^56^. MPV vaccine showed the most convenient basal profile of immune modulation regarding protection at PV1 showing the highest IL-4 expression, significant TNF and IFN-γ increases and lowest IL-10 expression. IL-10 has been described to have a role in immune tolerance^57^ and the blockage of IL-10 during immunization has demonstrated better pathogen clearance results in some studies^58^. The results of our study could support the idea that maintaining IL-10 levels low during immunization could be a good approach to avoid mucosal tolerance of Map.

Upon in vitro re-stimulation of PBMCs with Map sonicate (ex vivo secondary exposure), in general, an innate immune activation driving inflammatory response was characterized by increased IL-1β, TNF, and IL-23A expression, accompanied by an increase of the regulatory IL-10. This IL-10 elevation has also been reported for this vaccine in a study that involved in vitro infection of caprine monocyte-derived macrophages with Map^13^ and for a recombinant protein cocktail vaccine calves^59^. Regarding PTB, there are discrepancies, since both the presence^59^ and absence^60^ of IL-10 after in vitro estimulation with Map have been associated to protection. MPV was the only group showing high IL-12B (p40) and IL-23A (p19) levels. IL-12 gene expression and secretion have been associated to protection^59^. High levels of IL-23 should be desirable since it has been linked to bacterial burden reduction and maintenance of a Th17 CD4 population in Mtb vaccination^61^.

Primed IL-1β response by gut monocytes and DCs, is considered to enhance gut protection, due to its role in inflammatory cell recruitment^62^ and PBMC derived IL-1β participates in neutrophil recruitment and activation and inhibition of neutrophil apoptosis^40^. In our study, all vaccinated animals showed an increased IL-1β expression a month after vaccination (just before challenge) upon re-stimulation with Map sonicate. Furthermore, early IL-1β basal increase correlated with increased phagocytic activity of neutrophils. In this sense, the vaccine triggering highest basal levels of IL-1β was CPV.

The GALT cytokine expression profile at the end point of the experiment showed a discrete pro-inflammatory response in most of the challenged animals, including all vaccinated groups and CC. The subclinical nature of the infection model is probably contributing to this fact. CV showed the highest lymphoproliferative expression in comparison to the oral vaccines probably due to the administration route and the persistence of the granuloma generated at the injection site, which has shown to correlate in size to anti-PPA3 antibody levels in the previous works^19^.

There has been controversy on whether inactivated vaccines maintain their innate training effects, mainly because evidence from human clinical data has claimed that the beneficial non-specific effects would be limited to live vaccines^63^, as seen by comparison of BCG and gamma-irradiated BCG^64^. On the other hand, there is proof that a vaccine composed of cell wall components from *Mycobacterium phlei* in oil-emulsion was able to decrease overall mortality in feedlot cattle^65^, and also to improve survival after enterotoxigenic *E. coli* challenge in neonatal cattle^66^. In any case, we believe that this training effect in inactivated vaccines should be revised since we have demonstrated cross protective effects of heterologous inactivated vaccines against PTB in the present study. On the other hand, live-attenuated vaccines against PTB have also been orally tested in goats compared to CV^67^. In that study, some of the experimental vaccines showed low protective capacity in terms of reduction of bacterial burden in tissues, as has happened in our study. The authors claim that oral vaccination might not be appropriate for delivery of live attenuated vaccines against PTB in the goat model. Incorporating an adjuvant to these vaccines and fine dose and time tuning can also be considered.

Data presented here have been obtained in the rabbit infection model and should be confirmed in ruminants. The results regarding trained immunity mechanisms obtained in this model are most probably similar in ruminants and will include training of PMNs and monocytes as seen in this study and in that conducted by Arteche Villasol et al.^13^, respectively. As for protection, we assume that the rabbit oral challenge model is suitable for subcutaneous and oral vaccine screening because it is susceptible to Map^3,4^ and it is a good model of intestinal inflammatory conditions^68^ but these findings must be demonstrated in ruminants.

In conclusion, we show that oral vaccination with inactivated bacteria is able to activate mucosal immunity and that PMNs can be trained through oral and subcutaneous PTB vaccination as seen by the increased antimicrobial functions in circulating PMNs upon stimulation with Map and other non-related pathogens. Attending to the properties exerted by the presented vaccine prototypes, CPV has shown interesting characteristics enhancing unspecific, but protective, PMN activation levels. This immunostimulatory capacity of the CPV vaccine is not surprising since different *Corynebacterium* species have shown to be potent immunostimulants^69,70^. For this reason, it could be worth studying its effect as a formulation ingredient to be used in combination with the Quil-A adjuvant and Map bacilli, or as a complement in MPV formulation. Further future studies should involve assessing if these enhanced microbial effects are acquired by the entire PMN population or only by some specific subsets considering that PMNs can be heterogenous in phenotype and function. Also studying the potential bactericidal activity by PMNs should be further examined.

## Methods

### Ethics statement

All animal procedures were carried out following European, National, and Regional regulations on animals used in experimentation and other scientific purposes. The protocols were evaluated and approved by the Ethics Committee at NEIKER (NEIKER-OEBA-2018-0001) and authorized by the Regional Council (BFA-38012).

### Vaccine and bacteria inoculum preparation

All bacteria used for ex vivo and in vivo challenge assays were grown to the exponential phase at 37 °C under aerobic conditions.

#### Vaccines

The MPV vaccine (inactivated Map strain 316F vaccine) was prepared as described previously in Arrazuria et al.^19^. The CPV vaccine (inactivated *Corynebacterium pseudotuberculosis* field strain vaccine) was grown in brain heart infusion (BHI) until mid-exponential phase and was inactivated following the same inactivation protocol as that described for MPV^19^. The MBV vaccine (inactivated Mbv strain 1403 vaccine) was prepared as described in Garrido et al.^71^. The LAV vaccine (live attenuated vaccine) was created by allelic exchange mutagenesis as previously described^72^ and was grown on Middlebrook 7H9 broth supplemented with 10% OADC, 0.4% glycerol and 2 g/L mycobactin J (7H9 OADC MJ) with hygromycin (75 µg/ml) and shaking at 140 rpm until exponential phase (OD_600_ = 0.6–0.8).

#### Oral challenge

Bovine Map cattle field strain 764 used for challenge was grown on 7H9 OADC MJ for three weeks and adjusted to 1,5×10^8^ CFU/ml based on data from growth curves and plating assuming that 0.7 OD is 1 × 10^8^ bacteria/ml for Map.

#### Ex vivo assays

Map-K10-GFP (Map-GFP) and *M. bovis* (Mbv) strain 2008/2575 were grown for 3 weeks on 7H9 OADC MJ kanamycin (25 µg/ml) and 7H9 OADC, respectively. *Escherichia coli* (Ecoli)*, S. aureus* (Saur), and *C. pseudotuberculosis* (Cpstb) field strains were grown on BHI broth for 24 h in the case of *E. coli* and *S. aureus* and 48 h for *C. pseudotuberculosis*. Inocula were adjusted after measuring the optical density at 600 nm and based on data from growth curves and plating assuming that 0.7 OD is 1 × 10^8^ bacteria/ml for Map, Mbv and Cpstb, 0.4 OD is 2 × 10^8^ bacteria/ml for Saur and 0.7 OD is 5.6 × 10^8^ bacteria/ml for Ecoli.

### Experimental design

New Zealand White female rabbits of 7 weeks of age were purchased from authorized experimental animal dealers (Granja Cunícola San Bernardo, Tulebras, Spain) and left on a 15 day acclimatization period. A schematic representation of the experimental design is shown in Fig. 1. Thirty five rabbits were housed in cages being divided into seven groups of five animals each: non-vaccinated and non-challenged group (NC), non-vaccinated challenged group (CC), subcutaneously vaccinated group with the commercially available vaccine Silirum® (CV) and four orally vaccinated groups: live attenuated vaccine (LAV)^72^, inactivated Map strain 316F vaccine (MPV)^19^, inactivated Mbv strain 1403 vaccine (MBV) and inactivated *C. pseudotuberculosis* field strain vaccine (CPV). Before challenge, at initial time points, NC and CC, were considered as one group and named NV (non-vaccinated). The inactivated commercial vaccine was administered subcutaneously as a single dose (12.5 mg of antigen in 1 ml) on day 1. All orally administrated inactivated vaccines were formulated with Quil-A® adjuvant (InvivoGen) at 50 μg/dose. Oral vaccines (LAV, MPV, MBV, and CPV) were administered in two doses (12.5 mg of antigen per dose to match CV antigen quantity as calculated by wet weight following guidelines for PTB vaccination trials^73^ in 2 ml for LAV, MPV, and CPV (ranging 3.21–4.06 ×10^7^/dose) and 10^7^ CFU for MBV on days 1 and 15. On days 32, 33, and 34 all groups except for NC were challenged orally with one dose of 3 × 10^8^ CFU of Map strain 764 in 2 ml of PBS. Blood sampling for Map PPA-3 ELISA was performed monthly throughout the study. Neutrophils were isolated from blood for ET release assays on weeks 4 (PV1) and 12 (PV3) and for phagocytosis assays on weeks 4 (PV1), 12 (PV3), and 16 (PV4). Also, blood was extracted in heparin for autologous plasma used in phagocytosis assays on weeks 4 (PV1), 12 (PV3) and 16 (PV4). At the end point, 24 weeks after the start of the experiment, all animals were euthanized by intracardiac administration of pentobarbital (Vetoquinol) after deep sedation with xylazine (Calier) and ketamine (Merial). Tissue samples from the *sacculus rotundus* (SAC), vermiform appendix (VA), mesenteric lymph nodes (MLN), tongue, tonsils and cecal content from SAC were then collected and stored at −20 °C for Map isolation, since this was considered the experimental endpoint parameter.

### Lactate quantification assay

Lactate levels in plasma samples were quantified using the Lactate-Glo™ Assay (Promega) following the manufacturer’s instructions, using 50 μl of samples (1:100) and ten two-fold serial dilutions of a lactate standard run in duplicate in 96-well plates. Luminescence was recorded using a plate-reading luminometer (Promega). Lactate quantity was obtained extrapolating sample luminescence values from the standard curve.

### PBMC isolation

Seventeen ml of blood were collected from the auricular artery in Acid Citrate Dextrose vacutainer tubes. Red blood cells (RBCs) were eliminated by blood dilution 1:2 with 2.4% Dextran/0.9% NaCl. After mixing by inversion, tubes were left undisturbed for 30 min until two different phases were visible. The upper clearer layer, rich in leukocytes, was transferred to a 50 ml tube to pellet the cells by centrifugation at 300 × *g* for 10 min. After the supernatant was removed, 10 ml of rabbit leukocyte buffer (NaCl 138 mM, KCl 27 mM, Na_2_HPO_4_ 8.1 mM, KH_2_PO_4_ 1.5 mM, glucose 5.5 mM) was added to the pellet and the cell suspension was layered on top of 10 ml Histopaque® 1119 (Sigma-Aldrich) and centrifuged at 700 × *g* for 35 min at RT, with no brake. The PBMC layer localized between the saline and the Histopaque® 1119 solution was aspirated and transferred to a new tube for serial washing in saline. PBMCs were counted on a BIORAD TC20 counter and frozen in RPMI supplemented with FBS (40%) and DMSO (10%) at −80 °C until use.

### PMN isolation

PMNs were isolated from the same 17 ml of blood sample passed through Histopaque® 1119 used for PBMC isolation following a modified protocol from Siemsen et al.^74^. After the PBMC layer was aspirated and Histopaque® 1119 was eliminated, PMNs localized in the pellet together with remaining RBCs were aspirated to a new tube and went through hypotonic lysis with 3 ml of distilled water for 30 s. One ml of KCl 0.6 M was then added and the samples were centrifuged at 300 × *g* for 10 min. PMNs were suspended in rabbit leukocyte buffer and counted on a Bio-Rad TC20™ counter. This isolation protocol yielded PMNs with 94.4 ± 5.9% purity and 98.68 ± 1.1% viability (Supplementary Fig. 1).

### Neutrophil phagocytosis

Phagocytosis was performed in suspension following a previously reported method with modifications^75^. Briefly, freshly isolated PMNs suspensions were prepared in RPMI without phenol red supplemented with 2% FCS at 10^7^ cells/ml. Opsonization of bacteria was performed by incubation in rotation al 37 °C of 400 μl suspensions of a 1:1 dilution of heat-inactivated (52 °C, 15 min) autologous plasma in RPMI with L-glutamine and without phenol red (Gibco). Autologous opsonized and non-opsonized Map-GFP was added to vials with PMNs at multiplicity of infection (MOI) of 10 and gently mixed and incubated on a wheel shaker at 37 °C at 16 rpm for 20 min.

Live-Dead cells were determined by Hoechst 33258 (H3569, Invitrogen) staining at 0.025 mg/ml for 15 min at 4 °C. Cell suspensions were analyzed using a CytoFLEX Flow Cytometer (Beckman Coulter). Gating strategy is detailed in Supplementary Fig. 2. Data from the experiments were depicted as percentages of GFP positive PMNs of at least 10,000 events. Flow cytometry data were analyzed with CytExpert v2.3 software (Beckman Coulter). Phagocytosis percentages were expressed as the number of Map-GFP positive cells divided by the total number of live and single PMNs.

### Neutrophil extracellular trap (NET) release quantification

The protocol followed for NET release is a modification of Köckritz-Blickwede et al.^76^. Briefly, freshly isolated PMNs were seeded into 96-well plates at a density of 2 × 10^5^ cells/well. Stimulation was performed with PMA (Sigma-Aldrich) at 25 nM and *S. aureus*, *E. coli*, Cpstb, Mbv, and Map at MOI 5. Plates were incubated for 4 h at 37 °C 5% CO_2_. Non-stimulated PMNs were used as a negative control. The NETs generated by PMNs were digested with 500 mU/ml of s7 nuclease (Roche) for 10 min at 37 °C 5% CO_2_. Nuclease activity was stopped with 5 mM EDTA and culture supernatants were collected and stored at 4 °C ON. Total DNA was extracted from non-stimulated PMNs with DNazol supplemented with 1% polyacryl carrier (Molecular Research Center) following the manufacturer’s instructions. Extracted DNA was solubilized in TE buffer. Both NETs and genomic DNA was quantified using the Quant-iTTM PicoGreen® assay (Thermofisher) according to the manufacturer´s instructions. The plates were read in a fluorescence microplate reader (Synergy HTX, Biotek) with filter settings at 488 nm excitation and 520 nm emission. Data from controls with no cells and non-stimulated cells were subtracted. The percentage of released NET-DNA was calculated by dividing the amount of isolated NET-DNA by the DNA genomic content and NETosis levels were expressed as percentages.

### Neutrophil extracellular trap (NET) visualization

Visualization was performed on NETs developed on 16 well chamber slides (Thermo Scientific) in identical conditions as those described for NET quantification in the 96-well plates. After the 4 h incubation period, PMN preparations were fixed with formaldehyde 4% for 15 min. Two washes with PBS were performed and chambers were kept in PBS at 4 °C until staining was performed. Before staining, cells were permeabilized for 15 min with PBS 0.1% TRITON 100, blocked with PBS 1% Goat serum, 0.05% Tween 20, and 3% BSA. The immunofluorescence staining was performed with the mouse anti-pan-histone (Merk MAB3422) (1:200) ON at 4 °C. After 3 washing steps with PBS, cells were incubated with anti-mouse Alexa Fluor 594 (Invitrogen) (1:500) for 30 min at RT. After 3 washing steps, cover slips were mounted on the slides with a drop of mounting medium containing DAPI. Micrographs were taken on a fluorescence microscope (Leica-DMi8).

### Map culture sonication

Bovine Map cattle field strain 764 strain was sonicated and used for in vitro stimulation. Briefly, Map was grown in 7H9 OADC MJ broth for three weeks at 37 °C in aerobic conditions, to an optical density of 0.4 OD at 600 nm. The culture was centrifuged at 10,000 × *g* for 20 min and the pellet was washed twice with cold PBS. The pellet was suspended in PBS and sonicated three cycles of 5 min at 18 W on ice in a Vibra Cell 75186 sonicator. Protein concentration was determined on a spectrophotometer NanoDrop® and was adjusted to 100 µg/ml.

### PBMC stimulation

PBMCs were thawed, washed twice, and incubated in complete medium (RPMI 1640 with 10% FBS) at 37 °C and 5% CO_2_. The cells were then washed, counted, suspended in complete medium, and seeded at 1 × 10^6^ cells/well on 24 well plates and incubated 1 h at 37 °C and 5% CO_2_ prior to stimulation. Stimulation was performed with Concanavalin A (Sigma-Aldrich) (ConA) (2.5 µg/ml), Map sonicate (SM) (10 µg/ml) or left untreated (PBS). Cultures were incubated for 24 h at 37 °C °C and 5% CO_2_ and centrifuged at 300 × *g* for 8 min for RNA extraction.

### RNA isolation and conversion to cDNA of PBMCs and gut associated lymphoid tissue

RNA isolation from basal and stimulated PBMCs and gut associated lymphoid tissue (GALT; sacculus rotundus) was performed using the RNeasy® minikit (Qiagen). Briefly, RLT buffer supplemented with 2-mercaptoethanol was added to 5 × 10^6^ pelleted cells (350 µl) or to 20 mg of tissue (600 µl). Disruption and homogenization were performed on Tissuelyser II (Qiagen) for cells (one cycle of 30 Hz for 9 min) and tissues (two cycles of 20-30 Hz for 2 min with a stainless steel bead 7 mm per tube). All samples were stored at −80 °C until use. From this point, the RNA isolation procedure followed the manufacturer’s instructions. The RNA was treated with DNAse I (Invitrogen). DNAse treated RNA yield was quantified using a NanoDrop® spectrophotometer. All the samples were adjusted to 50 ng/ml RNA concentration and were reverse transcribed to cDNA.

cDNA synthesis and RT minus controls of RNA from GALT and PBMCs were performed on a final volume of reaction 10 µl and 20 ng/µl of RNA concentration. The reaction contained 4 µl of sample, 0.5 µl of Random Primers, 0.5 µl Oligo Dt, 0.75 µl of DEPC-water (1.25 µl for RT-controls), 2 µl of Buffer 5×, 1 µl of MgCl_2_, 0.5 µl of nucleotide mix, 0.25 µl of RNAsin, and 0.5 µl of GoScript ^TM^ reverse transcriptase (0 µl for RT- controls) using the GoTaq® 2-Step RT-qPCR System (Promega). The RT thermocycler program followed the manufacturer´s recommendation.

Primers for transcripts of IFN-γ, TNF, IL-2, IL1-β, IL-4, IL-12B, IL-10, and IL-23A cytokines and *IPO8, PGK1, RPLP0, UBE2D1, YWHAZ* reference genes were designed using the IDT online PrimerQuest Tool following MIQE guidelines^77^. Primer sequences are detailed on Supplementary Table 1.

### Cytokine expression level quantification

Fluidigm (Specific target amplification (STA) and qPCR using the BioMark^TM^ HD system) for cytokine expression level determination was performed at SGIker UPV-EHU. The commercially available Multiplex PCR kit (Qiagen) was used for the STA of the samples in a final reaction volume of 5 µl, containing 1.25 µl of the cDNA and 100 nM of primer concentration. The thermocycler program performed for STA consisted on a stage 1: 95 °C for 15 min and a stage 2:12 cycles at 95 °C for 15 s followed for 4 min at 60 °C, at the end, a final step at 4 °C ∞. Then, STA samples were treated with Exonuclease I (Thermo Scientific) adding 2 µl of a master mix (1.4 µl DEPC water, 0.2 µl Exonuclease I Reaction Buffer, 0.2 µl Exonuclease I (20 units/µl)), to each 5 µl of STA sample and the following program was applied: 37 °C for 30 min (Digestion), 80 °C for 15 min (Inactivation Exo I) and 4 °C ∞ (chill). Then STAs were diluted 1:20 in TE (10 mM Tris, 0.1 mM EDTA).

The expression analysis of the pre-amplified Exonuclease I-treated cDNA was carried out in the qPCR BioMark HD (Fluidigm) nanofluidic system in combination with 192.24 Dynamic Arrays IFC (IFC) (192 samples, 24 assays). SsoFast^TM^ EvaGreen® Supermix with Low ROX (Bio-Rad Laboratories). Final testing conditions for each pair of primers are detailed on Supplementary Table 1. The 9 target genes were migrated in duplicates. PCR (GE 192 × 24 Fast PCR + Melt v2) protocol was performed: Stage 1 (Hot start): 95 °C for 5 s, stage 2 (Ramp rate 5.5 °C/s): 30 cycles at 96 °C for 5 s and 60 °C for 20 s and stage 3 (Melting Curve): 60 °C for 3 s and from 60 °C to 95 °C (ramp rate slow 1 °C/3 s).

Cts (Cycle threshold) and Cqs (Quantification Cycle) results were analyzed using the Fluidigm Real-Time PCR Analysis Software version 4.1.3. Thresholds were manually adjusted for each primer pair. Apart from duplicates of STA samples, a no template control (NTC), 8 RT minus, and five serial 1/5 dilutions from a concentrated sample for the efficiency analysis were included in the run. The MultiD GenEx vs 6.1 software was used for calculating: qPCR efficiency, efficiency correction of Cts, replicate analysis and mean calculation, reference gene stabilization analysis, normalization of Cqs using the best reference genes and relative expression calculation using the comparative 2−ΔΔCT method with the efficiency correction. More information on the analysis for the selection of the best reference gene combinations for each sample type is detailed in Supplementary Figs. 2–4.

### Map PPA-3 ELISA

Homemade indirect ELISA was performed using Map protoplasmatic antigen 3 (PPA-3) (Allied Monitor) as previously described^78^. Briefly, plasma samples were adsorbed with *Mycobacterium phlei* in saline solution (5 g/l) at equal concentrations to reduce cross-reactivity. Microtiter plates were coated with 0.04 mg/mL of PPA-3 diluted in 0.5% sodium carbonate buffer (pH 9.6). Plasma samples were diluted 1:200 in 0.05% Tween 80 in PBS (PBS-T) and were assayed (100 µL/well) and protein G peroxidase (P8170, Sigma-Aldrich) at 0.025 μg/ml) was used. Absorbance was measured at 405 and 450 nm using an automated ELISA plate reader (Multiskan EX®). The reading obtained at 450 nm was subtracted from the reading of 405 nm to reduce optical imperfections in the plate. The results are expressed as a relative absorbance index calculated by dividing the mean absorbance of the sample by the mean absorbance of the negative control sample.

### Map isolation from tissues

For culture on Herrold’s Egg Yolk Medium (HEYM), 0.5 g of collected tissues were processed. Mesenteric lymph node was sliced in tiny pieces and weighed, whereas vermiform appendix and *sacculus rotundus* were scraped for collection of mucosa and weighed and samples were processed as described previously^22^. Briefly, hexadecylpyridinium chloride (Sigma-Aldrich) 0.76% decontaminated suspensions were centrifuged at 2885 × *g* during 10 min. The supernatant was discarded and the pellet was washed once with sterile water. After a new centrifugation step in the same conditions the pellet was suspended in 2 ml of water and four drops/tube were seeded. All seeded tubes were incubated at 37 °C and checked for MAP growth at 8, 12, 16, and 20 weeks.

For culture on 7H11 OADC PANTA TiKa MOPT^79^, 100 mg of collected tissues were sliced and macerated on a Stomacher®. Afterward, these were treated with TLB buffer supplemented with collagenase and trypsin, thoroughly mixed and incubated with shaking ON at 37 °C. The digested tissue was centrifuged at 14,000 × *g* for 10 min. The pellet was then re-suspended in 10 ml of TiKa-Kic (TiKa Diagnostics) and incubated for 24 h at 37 °C with gentle shaking. Samples were centrifuged at 10,000 × *g* for 15 min at RT. The pellet was re-suspended in 0.2 ml of sterile PBS and spotted onto 7H11 OADC PANTA TiKa-supplement A MOPT agar plates. Plates were incubated at 37 °C and checked for MAP growth at 4, 8, and 12 weeks.

Colonies isolated on both medium were confirmed by a real time multiplex PCR detecting *IS900* and *ISMap02* Map sequences^80^. In order to rule out LAV growth in tissues instead of the Map challenging strain 764, tissues from animals belonging to the LAV vaccinated group were also seeded on 7H9 OADC MJ with hygromycin (75 µg/ml).

### Statistical analyses

The data were assessed for normal distribution with the normality tests Shapiro–Wilk and Lilliefors (Kolmogorov–Smirnov). Homogeneity of variances was assessed with Bartlett’s test. For the statistical analysis of comparison between means of variables with a normal distribution, Student’s *t* test or ANOVA was used with post-hoc multiple comparison as follows: ET release, Tukey HSD; peripheral and sonicate treated RT-qPCR, pooled SD and BH correction, GALT RT-qPCR, Tukey HSD. For variables with a non-normal distribution, the Mann–Whitney *U* or Kruskal–Wallis tests were used with post-hoc multiple comparison as follows: phagocytosis, Dunn’s test with BH correction; Lactate, Dunn’ test. Pearson (ρx,y) coefficient was calculated for correlation between variables.

At PV1, sampling is performed before challenge and therefore NV control is used as a sum of CC and NC, as at this time of the experiment both groups are non-vaccinated groups.

The efficiency of the amplification of each pair of primers was calculated from the slope of the line resulting from the linear regression of the serial dilutions GenEx software corrects the raw Cqs using the efficiencies. Reference Gene stability analysis and normalization were also performed using the GenEx software, which uses NormFinder^81^ and GeNorm^82^ algorithms. For normalization, the geometric mean of the best combination of reference gene Cqs of each sample was subtracted to each sample Cq for every target gene.

2−ΔΔCT quantification method was performed with respect to the NC group for basal PBMC and *sacculus rotundus* expression, and with respect to non-stimulated PBMCs for stimulated PBMCs. The logarithmic transformation of the relative quantities Log2 RQ has been used as input for Fold change, calculation statistical analysis and plot and graph representation. The absolute Fold Changes were calculated using the expression: log2Fold change.

For the PCA analysis the following parameters were taken into account: phagocytosis of opsonized and non-opsonized Map-GFP at PV1, PV3, and PV4; NETosis percentages against Map, Cpstb, Mbv, Ecoli, and Saur at PV1 and PV3; anti-PPA3-IgG/IgM ELISA results at PV1, PV2, PV3, PV4, and PV5; total serum IgA levels at PV1; plasma lactate levels at PV1, PV2, and PV3; expression of all assayed cytokines in PBMCs at PV1; expression of all assayed cytokines in PBMCs stimulated with Map sonicate for 24 h at PV1 and expression of all assayed cytokines in GALT at EP of the experiment.

All the statistical tests were performed using R studio desktop (version 1.2.5033). (RStudio Team (2019). RStudio: Integrated Development for R. RStudio, Inc., Boston, MA URL http://www.rstudio.com/). A *p*-value <0.05 was considered statistically significant.

### Reporting summary

Further information on research design is available in the Nature Research Reporting Summary linked to this article.

## Supplementary information

Supplementary Files

Reporting summary

## Data Availability

The datasets generated for this study are available on request. The raw data supporting the conclusions of this article will be made available by the authors, without undue reservation.

## Code availability

All the codes used in R software for the analysis of the date for this study are available on request.
