# Author Correction: Oral vaccination stimulates neutrophil functionality and exerts protection in a *Mycobacterium avium* subsp. *paratuberculosis* infection model

**DOI:** 10.1038/s41541-026-01380-5

**Published:** 2026-02-06

**Authors:** Iraia Ladero-Auñon, Elena Molina, Maddi Oyanguren, Diego Barriales, Miguel Fuertes, Iker A. Sevilla, Lucy Luo, Rakel Arrazuria, Jeroen De Buck, Juan Anguita, Natalia Elguezabal

**Affiliations:** 1 Animal Health Department, Basque Institute for Agricultural Research and Development, NEIKER- Basque Research and Technology Alliance (BRTA), Derio, Bizkaia Spain; 2 Food Quality and Safety Department, Universidad del País Vasco/Euskal Herriko Unibertsitatea (UPV/EHU), Vitoria, Araba Spain; 3 Inflammation and Macrophage Plasticity Laboratory, CIC bioGUNE-Basque Research and Technology Alliance (BRTA), Derio, Bizkaia Spain; 4 Department of Production Animal Health, Faculty of Veterinary Medicine, University of Calgary, Calgary, AB Canada; 5 Ikerbasque, Basque Foundation for Science, Bilbao, Spain

**Keywords:** Inactivated vaccines, Innate immunity

Correction to: *npj Vaccines* 10.1038/s41541-021-00367-8, published online 12 August 2021

In the original Article, Figure 7 was inadvertently published twice, appearing also as Figure 8. The correct Figure 8 and its corresponding figure legend is provided below. The original Article has been corrected.

Incorrect Figure 8
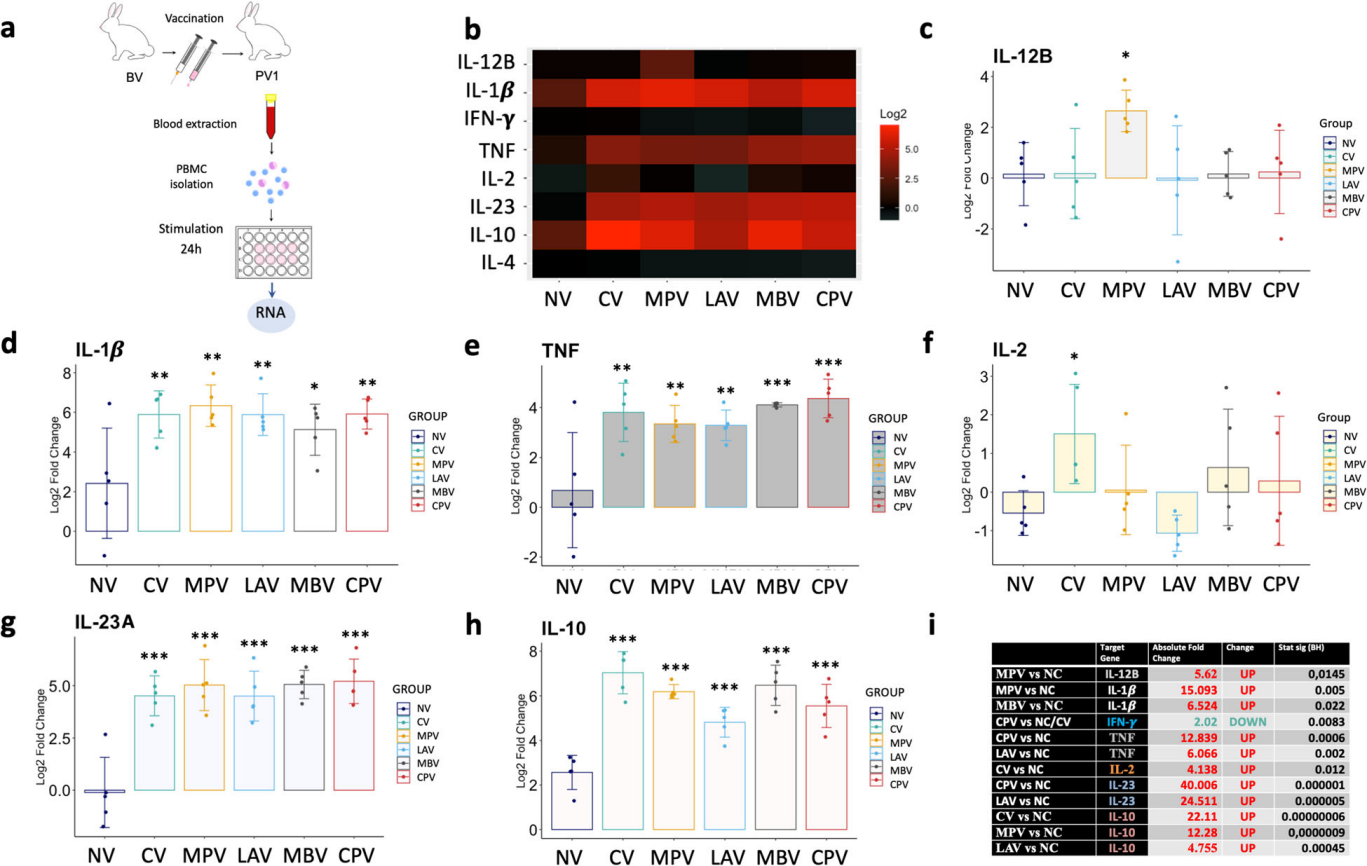


Incorrect Figure 8 legend:

**Cytokine relative quantification by RT-qPCR in GALT at the end point of the experiment of all challenged groups relative to the NC. a** Schematic representation of sample origin. **b** Heatmap representation of the average expression of cytokines; in red upregulated and in green downregulated gene expression. Bar charts showing log2 fold change between groups. **c** IL-12B, **d** IL-1β, **e** TNF, **f** IL-2, **g** IL-23A, **h** IL-10, **i** Table summarizing significant differences between groups in cytokine expression and the Absolute Fold Change Factor. All values were means with error bars representing standard deviation from groups of *n* = 5, except for NC which was *n* = 4. ANOVA with Tukey’s post-host test was applied and signification levels are *p < 0.05, **p < 0.01, ***<0.001.

Correct Figure 8
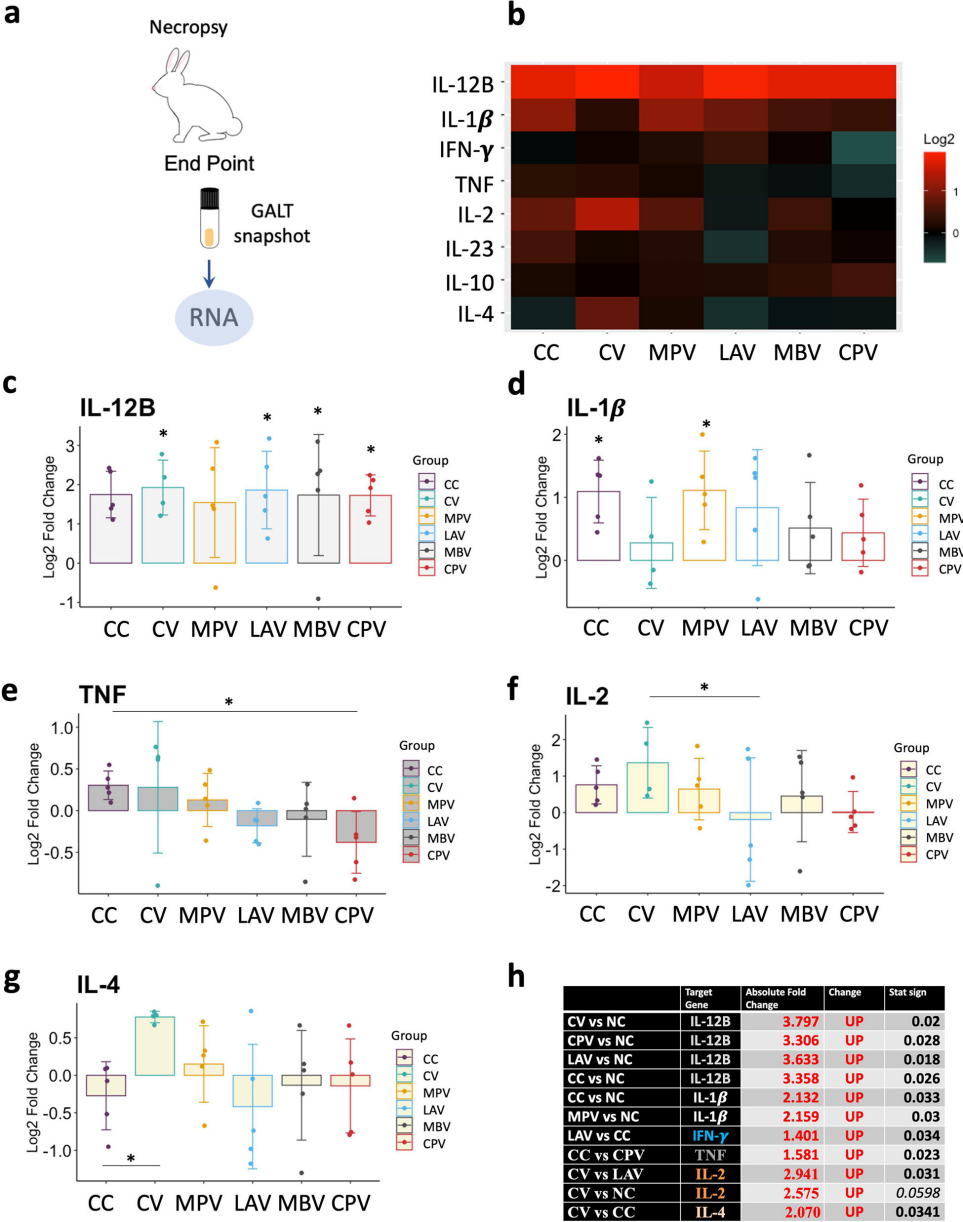


Corrected Figure 8 legend:

Cytokine relative quantification by RT-qPCR in GALT at the end point of the experiment of all challenged groups relative to the NC. **a** Schematic representation of sample origin. **b** Heatmap representation of the average expression of cytokines; in red up-regulated and in green down-regulated gene expression. Bar charts showing log2 fold change between groups. **c** IL-12B, **d** IL-1b, **e** TNF, **f** IL-2, **g** IL-4, **h** table summarizing significant differences between groups in cytokine expression and the Absolute Fold Change Factor. All values were means with error bars representing standard deviation from groups of *n* = 5, except for NC which was *n* = 4. ANOVA with Tukey’s post-host test was applied and signification levels are *p <0.05, **p <0.01, ***p <0.001.

