# Supplementary material for: Oral vaccination stimulates neutrophil functionality and exerts protection in a Mycobacterium avium subsp. paratuberculosis infection model
Source: NPJ Vaccines. 2021 Aug 12;6:102. doi: 10.1038/s41541-021-00367-8 (PMC8361088; doi:10.1038/s41541-021-00367-8)
Supplement: Supplementary file 1 — Supplementary Files [file 41541_2021_367_MOESM1_ESM.pdf]

Oral vaccination stimulates neutrophil functionality and exerts protection in a *Mycobacterium avium* subsp. *paratuberculosis* infection model

Iraia Ladero-Auñon[1,2], Elena Molina[1], Maddi Oyanguren[1], Diego Barriales[3], Miguel Fuertes[1], Iker A Sevilla[1], Lucy Luo[4], Rakel Arrazuria[4], Jeroen De Buck[4], Juan Anguita[3,5], Natalia Elguezabal[1*]

[1]Animal Health Department, Basque Institute for Agricultural Research and Development, NEIKER- Basque Research and Technology Alliance (BRTA), (Derio) Bizkaia, Spain
[2]Food Quality and Safety Department, Universidad del País Vasco/Euskal Herriko Unibertsitatea (UPV/EHU), (Vitoria) Araba, Spain
[3]Inflammation and Macrophage Plasticity Laboratory, CIC bioGUNE-Basque Research and Technology Alliance (BRTA), (Derio) Bizkaia, Spain
[4]Department of Production Animal Health, Faculty of Veterinary Medicine, University of Calgary, Calgary, AB T2N1N4, Canada
[5]Ikerbasque, Basque Foundation for Science, Bilbao, Spain

*Corresponding author: nelguezabal@neiker.eus

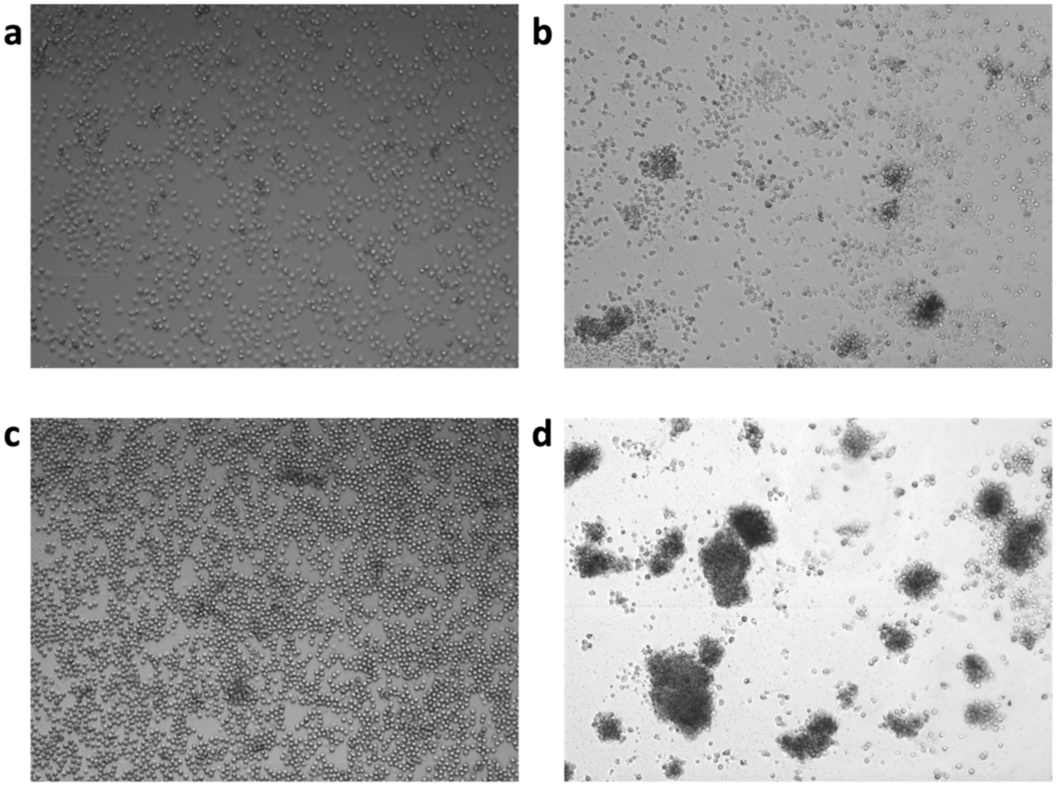

Supplementary Figure 1. Light microscopy micrographs (10x) of PMN preparations at PV1 of NV (a-b) and CV (c-d) stimulated with PMA (a, c) and Map (b, d).

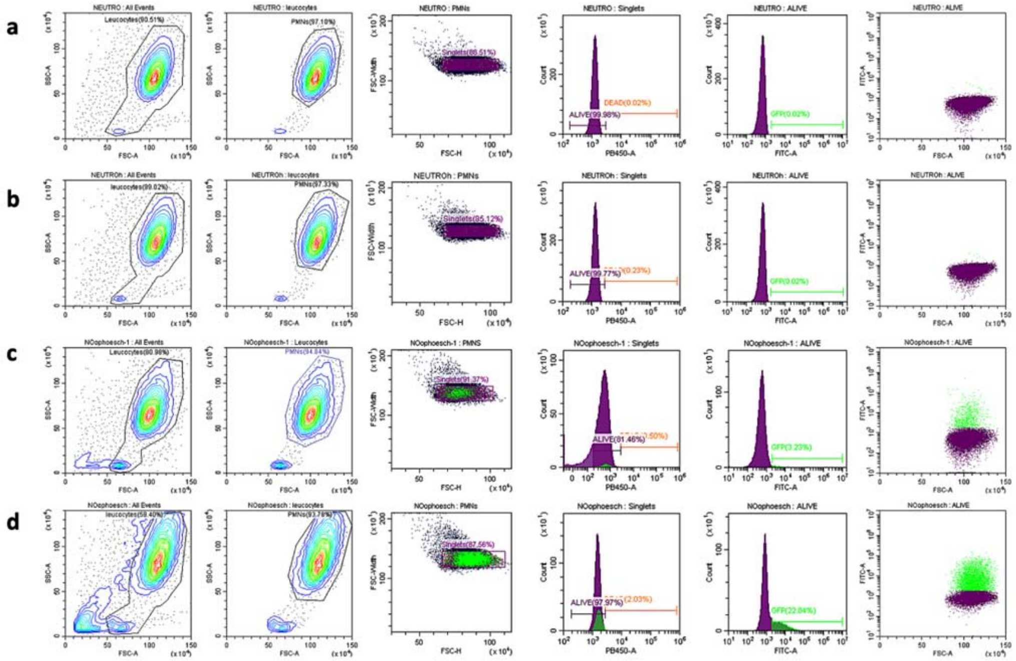

Supplementary Figure 2. Gating strategy used for the phagocytosis analysis. (a) PMNs without live-dead staining and without Map-GFP, (b) PMNs with live-dead staining and without Map-GFP, (c) PMNs of the NC group with live-dead staining and with Map-GFP, (d) PMNs of the NC group with live-dead staining and with Map-GFP. In the first column details about leukocyte gating based on FSC and SSC; in the second step PMN-gating based on FSC and SSC (purity information); in the third column doublet discrimination strategy; in the fourth column dead cell discrimination strategy by fluorescence in the PB450 channel; in the fifth column GFP associated PMN selection by fluorescence in the FITC channel and in the sixth column a scatter plot based on FITC-FSC of single-alive-PMNs.

a **NORMFINDER**

INTRA-GROUP VARIATION

GROUP	RPLP0	PGK1	IPO8	UBE2D1	YWHAZ
1	0,1683	0,1042	0,0302	0,0353	0,0058
2	0,0122	0,0449	0,0067	0,0135	0,0041
3	0,0464	0,0873	0,0378	0,008	0,0059
4	0,0034	0,0397	0,073	0,0279	0,0076
5	0,0112	0,0257	0,0363	0,0323	0,0058
6	0,4562	1,7302	0,4972	0,014	0,014
7	0,0189	0,0102	0,0066	0,0215	0,0054

INTER-GROUP VARIATION

GROUP	RPLP0	PGK1	IPO8	UBE2D1	YWHAZ
1	0,0741	-0,1965	0,1579	0,1464	-0,1819
2	-0,0754	0,0805	0,0968	0,0003	-0,1022
3	-0,026	0,024	-0,1301	-0,0969	0,229
4	-0,1089	0,0837	0,1206	-0,0144	-0,081
5	-0,031	0,1238	-0,1462	-0,0216	0,0749
6	0,1406	-0,2587	0,0242	-0,0896	0,1835
7	0,0266	0,1432	-0,1232	0,0757	-0,1222

b

Best gene: YWHAZ, SD: 0,0592

Best Combination of two genes: YWHAZ + UBE2D1, SD: 0,0492

Gene Name	SD	Acc. SD	SD	0,1993
YWHAZ	0,1993	0,1993		
RPLP0	0,2054	0,1431	Best Gene	YWHAZ
IPO8	0,234	0,1232		
UBE2D1	0,2738	0,115		

BEST NUMBER OF GENES 4: YWHAZ, RPLP0, IPO8 AND UBE2D1

c

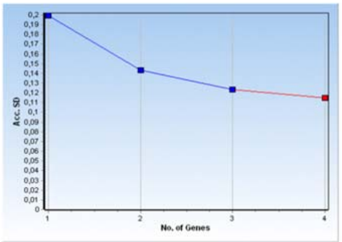

d **GENORM**

Gene Name	M-Value
PGK1	0,41026624
UBE2D1	0,3222696
YWHAZ	0,29957556
RPLP0	0,2594786
IPO8	0,2594786

Supplementary Figure 3. Best reference gene combination analysis for normalization of RT-qPCR expression in PBMC samples at PV1 (a) NormFinder Intra-group and inter-group variation results. PGK1 gene showed high intra-group variation (>1) in the group 6. All the genes showed low inter-group variation. (b) NormFinder provided best gene, best combination of two genes and best number of reference genes information after elimination of PGK1, the less stable gene. (c) NormFinder graph representation of the best number of reference genes for normalization. (d) GeNorm analysis results. In this case all five genes showed high stability M values (<0.5). UBE2D1, YWHAZ, RPLP0 and IPO8 resulted the most suitable combination of reference genes.

a **NORMFINDER**

INTRA-GROUP VARIATION

GROUP	RPLP0	PGK1	IPO8	UBE2D1	YWHAZ
8	0,0352	0,7615	0,1016	0,0972	0,0127
9	0,0042	0,7171	0,0297	0,7916	0,0042
10	0,0113	0,5967	0,0703	0,0078	0,0938
11	0,0114	0,367	0,038	0,258	0,0025
12	0,0173	1,5236	0,2161	0,2885	0,0032
13	0,0011	0,0888	0,015	0,285	0,0011
14	0,0158	0,048	0,1075	0,1888	0,0043
15	0,0022	0,4484	0,0219	0,1843	0,0158
16	0,0051	0,3938	0,0139	0,6346	0,0038
17	0,0081	0,2347	0,1658	0,1034	0,0011
18	0,0013	0,3623	0,0047	0,2272	0,0047
19	0,02	0,8371	0,0074	0,3378	0,0833
20	0,0044	0,3953	0,0703	0,2661	0,0639
21	0,0495	0,2595	0,0248	0,0636	0,0142
22	0,0243	0,2453	0,2022	0,3008	0,0313
23	0,005	0,1625	0,0282	0,5198	0,009
24	0,0196	0,2367	0,0095	0,3069	0,0658
25	0,0545	0,2687	0,0178	0,3248	0,0003
26	0,0085	0,4509	0,0425	0,3538	0,0125
27	0,0096	0,6041	0,5031	0,1621	0,0052
28	0,0247	0,3973	0,0019	0,1454	0,0077

INTER-GROUP VARIATION

GROUP	RPLP0	PGK1	IPO8	UBE2D1	YWHAZ
8	-0,0345	0,1349	-0,0017	0,0567	-0,1554
9	-0,1554	0,1091	-0,1506	0,2247	-0,0279
10	0,0221	0,009	0,2172	-0,1455	-0,1028
11	0,0673	0,266	0,0822	-0,3979	-0,0176
12	-0,0304	0,4033	0,007	-0,1862	-0,1938
13	-0,1709	0,1903	0,0789	-0,0075	-0,0908
14	0,3102	-0,3317	0,0803	-0,1489	0,0902
15	0,1004	0,0473	-0,0705	-0,0155	-0,0617
16	-0,0978	-0,1561	-0,111	0,3094	0,0556
17	-0,0063	-0,2581	0,1219	0,0071	0,1353
18	0,1855	0,1967	-0,3319	-0,1437	0,0934
19	0,0266	0,2798	-0,1337	-0,1091	-0,0636
20	0,0299	0,5059	-0,2628	-0,0961	-0,1768
21	0,1646	-0,2854	-0,0399	0,038	0,1226
22	0,1137	0,028	0,0742	-0,1212	-0,0947
23	-0,1999	-0,6469	0,0868	0,5905	0,1696
24	-0,1565	-0,4701	0,2154	0,167	0,2442
25	0,0217	-0,2095	0,0532	0,0053	0,1293
26	-0,1587	-0,1889	0,1523	0,126	0,0692
27	-0,1627	0,4748	-0,0076	-0,0856	-0,2189
28	0,131	-0,0982	-0,0598	-0,0675	0,0945

b

Best gene: YWHAZ, SD: 0,1607

Best Combination of two genes: YWHAZ + RPLP0, SD: 0,1356

Gene Name	SD	Acc. SD	SD	0,1384
YWHAZ	0,1384	0,1384		
RPLP0	0,2403	0,1386	Best Gene	YWHAZ
IPO8	0,288	0,1333		
UBE2D1	0,4952	0,1591		

Best number of genes: 3: YWHAZ, RPLP0 AND IPO8

c

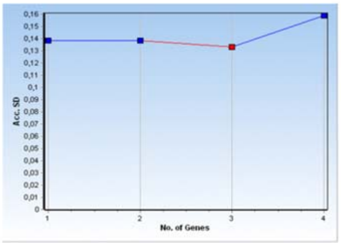

d **GENORM**

Gene Name	M-Value
PGK1	0,5452308
UBE2D1	0,41544932
IPO8	0,30437022
RPLP0	0,27670814
YWHAZ	0,27670814

Supplementary Figure 4. Best reference gene combination analysis for normalization of RT-qPCR expression in PBMC samples at PV1 after 24h stimulation, (a) NormFinder Intra-group and inter-group variation results. PGK1 gene showed a high intra-group variation (>1) in the group 12 and the highest inter-group variation, (b) NormFinder provided information about best gene, best combination of two genes and best number of reference genes after elimination of PGK1, the less stable gene, (c) NormFinder graph representation of the best number of reference genes for normalization, (d) GeNorm analysis results. PGK1 gene showed insufficient stability M value (>0.5). YWHAZ, RPLP0 and IPO8 resulted the best combination of reference genes.

a **NORMFINDER**

INTRA-GROUP VARIATION

GROUP	RPLP0	PGK1	IPO8	UBE2D1	YWHAZ
1	0,054	0,0317	0,1988	0,0034	0,001
2	0,0187	0,0086	0,03	0,0249	0,0233
3	0,0459	0,031	0,05	0,0256	0,0048
4	0,0458	0,0144	0,1639	0,0045	0,0062
5	0,0397	0,0663	0,1354	0,0175	0,0056
6	0,0178	0,0162	0,0518	0,0076	0,0237
7	0,0233	0,0564	0,0531	0,0033	0,0037

INTER-GROUP VARIATION

GROUP	RPLP0	PGK1	IPO8	UBE2D1	YWHAZ
1	0,126	0,0637	-0,4896	0,1949	0,105
2	-0,1915	-0,0592	0,3374	0,0344	-0,1211
3	-0,0716	-0,0128	0,0796	0,0137	-0,0089
4	0,031	0,0167	0,0288	-0,0661	-0,0103
5	-0,1075	-0,0124	0,1359	0,0109	-0,0269
6	-0,0183	-0,0126	-0,0173	-0,0528	0,1011
7	0,2319	0,0167	-0,0748	-0,1349	-0,0389

b

Best gene: YWHAZ, SD: 0,1067

Best Combination of two genes: YWHAZ + PGK1, SD: 0,1023

Gene Name	SD	Acc. SD	SD	0,0815
YWHAZ	0,0815	0,0815		
UBE2D1	0,1202	0,0726	Best Gene	YWHAZ
PGK1	0,1424	0,0678		
RPLP0	0,223	0,0755		
IPO8	0,3968	0,0997		

BEST NUMBER OF GENES: 3, YWHAZ, UBE2D1 Y PGK1

c

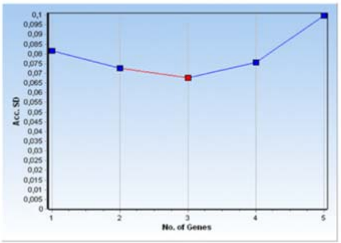

d **GENORM**

Gene Name	M-Value
IPO8	0,28872698
UBE2D1	0,20994529
RPLP0	0,18509162
PGK1	0,17329171
YWHAZ	0,17329171

Supplementary Figure 5. Best reference gene combination analysis for normalization of RT-qPCR expression in GALT samples. (a) NormFinder Intra-group and inter-group variation results. Al five genes are highly stable, (b) NormFinder provided information about stability values not considering groups, best reference gene, best combination of two genes and best number of reference genes, (c) NormFinder graph representation of the best number of reference genes for normalization, (d) GeNorm analysis results. In this case all five genes showed high stability M<0.5. Although the best number of recommended reference genes was three, as the difference in their M values was small, the normalization was performed with YWHAZ, PGK1, UBE2D1 and RPLP0.

Supplementary Table 1. Primer sequences for each gene, amplicon characteristic and PCR conditions.

Supplementary Table 1. Primer sequences for each gene, amplicon characteristic and PCR conditions.

Target gene (amplicon size)	Sequences	TmºC	GC%	PC (μM)
IFN-γ	5′TACTTTGGCATGGACTGTATT3′	58.6	38	0.7
(122)	5′CATAAAGCATGGTGGAGAA3′	58.7	42.1	0.7
IL-1β	5′CTCCTGCCAACCCTACAACA3′	57.1	55	0.3
(152)	5′GGACGGGTTCTTCTTCAA3′	52,4	50	0.3
IL-2	5′TATATGCCCAAGAAGGTCACA3′	61.9	42.8	0.4
(205)	5′TGGTAACTGTCTCATCGTATTCAC3′	61.8	41.6	0.4
IL-4	5′CAGTTCTACCTCCACCACAAG3′	61.2	52.3	0.2
(90)	5′GTTCCTGTCGAGTCCTCTC3′	58.9	57.8	0.2
IL-10	5′GCGACAATGTCACCGATTT3′	63.6	47.3	0.7
(98)	5′TGTAGACGCCTTCCTCTTG3′	60.6	52.6	0.7
IL-12A	5′CATCAGAGACATCATCAAACCT3′	60.6	40.9	0.5
(64)	5′GGAATTCTTCAATGGCTTCA3′	61.6	40	0.5
TNF	5′CTAGCCCACGTAGTAGCAAAC3′	60.4	52.3	0.5
(154)	5′AGAGAACCTGGGAGTAGATGAG3′	60.3	50	0.5
IL23A	5′CAGCAGCTCTCCCAGAA3′	56.2	58.8	0.5
(170)	5′AACTGAGTGTTGTCCCTTAGTC3′	55.8	45.5	0.3
RPLP0	5′CGTGAGAGTGACATCGTCTTTA3′	61.9	45.4	0.3
(111)	5′GGATGATCTTAAGGAAGTAGTTGGA3′	62.3	40	0.3
PGK1	5′TGGGCAAGGATGTTCTGTT3′	63.2	47.3	0.4
(112)	5′ACATGAAAGCGGAGGTTCT3′	61.3	47.3	0.4
YWHAZ	F 5′GCCCTTAACTTCTCTGTGTTCT3′	60.4	45.4	0.4
(136)	5′GCGTGCTGTCTTTGTATGATTC3′	63.5	45.4	0.4
IPO8	5′AGCTAGATCGTGCTGGGTA3′	60	52.6	0.3
(100)	5′GATCAGGCTCTTCTTGGCTAAT3′	62.5	45.4	0.2
UBE2D1	F 5′CCTGTGGGAGATGACTTGTTC3′	63.5	52.3	0.5
(82)	R 5′GGAAGAAGACTCCACCTTGATA3′	61.1	45.4	0.5

Tm: primer melting temperature; PC: primer concentration. For primer design, exon-exon junctions were taken into account, with at least one of each primer pairs between two exons. Target transcript sequences were obtained from the NCBI database. Dimers and hairpins were checked with IDT Oligo Analyzer Tools (https://eu.idtdna.com/site/account/login?returnurl=%2Fcalc%2Fanalyzer) and those with high possibilities of conforming secondary structures were discarded. Primer selection parameters were set to be: no more than 3 Gs or Cs in the last five nucleotides of the 3'end, CG% between 40-60%, primer size between 17-25 nts, Tm differences between primer pairs of less than 2ºC and product size 100-200 bp but down to 64 in a case where no suitable primers could be identified. Primers were ordered from Sigma-Aldrich and each pair was cross-tested for optimal working concentrations ranging 0.2-0.7 μM.
