# Supplementary material for: Oral vaccination stimulates neutrophil functionality and exerts protection in a Mycobacterium avium subsp. paratuberculosis infection model
Source: NPJ Vaccines. 2021 Aug 12;6:102. doi: 10.1038/s41541-021-00367-8 (PMC8361088; doi:10.1038/s41541-021-00367-8)
Supplement: Supplementary file 2 — Reporting summary [file 41541_2021_367_MOESM2_ESM.pdf]

nature portfolio

Corresponding author(s): DBPR-npjvaccines-00137R

Last updated by author(s): Jun 14, 2021

Reporting Summary

Nature Portfolio wishes to improve the reproducibility of the work that we publish. This form provides structure for consistency and transparency in reporting. For further information on Nature Portfolio policies, see our Editorial Policies and the Editorial Policy Checklist.

Statistics

For all statistical analyses, confirm that the following items are present in the figure legend, table legend, main text, or Methods section.

n/a	Confirmed	
☐	☒	The exact sample size (*n*) for each experimental group/condition, given as a discrete number and unit of measurement
☒	☐	A statement on whether measurements were taken from distinct samples or whether the same sample was measured repeatedly
☐	☒	The statistical test(s) used AND whether they are one- or two-sided<br>*Only common tests should be described solely by name; describe more complex techniques in the Methods section.*
☐	☒	A description of all covariates tested
☐	☒	A description of any assumptions or corrections, such as tests of normality and adjustment for multiple comparisons
☐	☒	A full description of the statistical parameters including central tendency (e.g. means) or other basic estimates (e.g. regression coefficient) AND variation (e.g. standard deviation) or associated estimates of uncertainty (e.g. confidence intervals)
☐	☒	For null hypothesis testing, the test statistic (e.g. *F*, *t*, *r*) with confidence intervals, effect sizes, degrees of freedom and *P* value noted<br>*Give P values as exact values whenever suitable.*
☒	☐	For Bayesian analysis, information on the choice of priors and Markov chain Monte Carlo settings
☒	☐	For hierarchical and complex designs, identification of the appropriate level for tests and full reporting of outcomes
☒	☐	Estimates of effect sizes (e.g. Cohen's *d*, Pearson's *r*), indicating how they were calculated

Our web collection on statistics for biologists contains articles on many of the points above.

Software and code

Policy information about availability of computer code

Data collection	Data was collected and set up in Microsoft Excel 2010 and saved in .xls and afterwards imported into R for analysis.
Data analysis	R graph and statistical analysis packages: ggplot2, ggpubr, nortest, Rmisc, PMCMR, ez, corrplot, graphics. Other R packages: PMCMRplus, plyr, dplyr, PMCMR, tidyverse, DescTools, scales.

For manuscripts utilizing custom algorithms or software that are central to the research but not yet described in published literature, software must be made available to editors and reviewers. We strongly encourage code deposition in a community repository (e.g. GitHub). See the Nature Portfolio guidelines for submitting code & software for further information.

Data

Policy information about availability of data

All manuscripts must include a data availability statement. This statement should provide the following information, where applicable:

- Accession codes, unique identifiers, or web links for publicly available datasets
- A description of any restrictions on data availability
- For clinical datasets or third party data, please ensure that the statement adheres to our policy

The datasets generated for this study are available on request. The raw data supporting the conclusions of this article will be made available by the authors, without undue reservation.

Field-specific reporting

Please select the one below that is the best fit for your research. If you are not sure, read the appropriate sections before making your selection.

☒ Life sciences ☐ Behavioural & social sciences ☐ Ecological, evolutionary & environmental sciences

For a reference copy of the document with all sections, see nature.com/documents/nr-reporting-summary-flat.pdf

Life sciences study design

All studies must disclose on these points even when the disclosure is negative.

Sample size	No sample-size calculation was performed. Experimental groups based on 4-5 animals for the PTB infection model have been used before obtaining statistically significant results (Arrazuria et al Res Vet Sci 2015, Arrazuria et al. Vet Res 2016, Arrazuria et al Front Microbiol 2016 and Arrazuria et al Vet Sci 2020)
Data exclusions	No data were excluded from the analyses.
Replication	The experimental infection model has shown to be reproducible Arrazuria et al Res Vet Sci 2015, Arrazuria et al. Vet Res 2016, Arrazuria et al Front Microbiol 2016 and Arrazuria et al Vet Sci 2020). All protocols are standarized and documented in the Research Institute's Quality and management system ( ). Animals, samples and reagents are also tracked. NEIKER has an R&D&I management system in compliance with Standard UNE 166002:2014 for research, development and innovation activities in the field of agri-food (agricultural, livestock and forestry sectors) and in the natural environment. Number: IDI-0009/2019 ,ISO 9001 Certification for the management of R&D projects and technology transfer since 2005 – ER-1201-2005 and Accreditation no. 615/LE 1321 from ENAC for the carrying out of physical and chemical, microbiological, immunological and molecular tests on agri-food products at the Department of Animal Health.
Randomization	Allocation of organisms to experimental groups was random. All animals belonged to the same lot, with similar characteristics.
Blinding	All organisms were identified with a number that was linked to a experimental group. Map tissue culture, IgA levels and PPA3 ELISA were blinded. Fluidigm service only had knowledege about organisms assigned to the non-challenged group. The rest of the studied parameters were performed by researchers that knew which experimental group they were analyzing at each time.

Reporting for specific materials, systems and methods

We require information from authors about some types of materials, experimental systems and methods used in many studies. Here, indicate whether each material, system or method listed is relevant to your study. If you are not sure if a list item applies to your research, read the appropriate section before selecting a response.

Materials & experimental systems

n/a	Involved in the study	
☒	☐	Antibodies
☒	☐	Eukaryotic cell lines
☒	☐	Palaeontology and archaeology
☐	☒	Animals and other organisms
☒	☐	Human research participants
☒	☐	Clinical data
☒	☐	Dual use research of concern

Methods

n/a	Involved in the study	
☒	☐	ChIP-seq
☐	☒	Flow cytometry
☒	☐	MRI-based neuroimaging

Animals and other organisms

Policy information about studies involving animals; ARRIVE guidelines recommended for reporting animal research

Laboratory animals	New Zealand White rabbits females of 7 weeks of age at beginning of experiment
Wild animals	*Provide details on animals observed in or captured in the field; report species, sex and age where possible. Describe how animals were caught and transported and what happened to captive animals after the study (if killed, explain why and describe method; if released, say where and when) OR state that the study did not involve wild animals.*
Field-collected samples	*For laboratory work with field-collected samples, describe all relevant parameters such as housing, maintenance, temperature, photoperiod and end-of-experiment protocol OR state that the study did not involve samples collected from the field.*
Ethics oversight	All animal procedures were carried out following European, National and Regional regulations on animals used in experimentation and other scientific purposes. The protocols were evaluated and approved by the Ethics Committee at NEIKER (NEIKER-OEBA-2018-0001) and authorized by the Regional Council (BFA-38012).

Note that full information on the approval of the study protocol must also be provided in the manuscript.

Flow Cytometry

Plots

Confirm that:

☒ The axis labels state the marker and fluorochrome used (e.g. CD4-FITC).

☒ The axis scales are clearly visible. Include numbers along axes only for bottom left plot of group (a 'group' is an analysis of identical markers).

☒ All plots are contour plots with outliers or pseudocolor plots.

☒ A numerical value for number of cells or percentage (with statistics) is provided.

Methodology

Sample preparation	PMNs were isolated fresh from Acid Citrate Dextrose peripherla blood by dextran sedimentation and density gradient on histopaque. Suspensions were prepared in RPMI without phenol red supplemented with 2% FCS at 107 cells/ml.
Instrument	Beckman Coulter Cytoflex
Software	Beckman Coulter CytExpert v2.3 software
Cell population abundance	The isolation protocol yielded PMNs with 94.4 ± 5.9 % purity and 98.68 ± 1.1 % viability
Gating strategy	Gating strategy used for the phagocytosis analysis. (a) PMNs without live-dead staining and without Map-GFP, (b) PMNs with live-dead staining and without Map-GFP, (c) PMNs of the NC group with live-dead staining and with Map-GFP, (d) PMNs of the NC group with live-dead staining and with Map-GFP. In the first column details about leukocyte gating based on FSC and SSC; in the second step PMN-gating based on FSC and SSC (purity information); in the third column doublet discrimination strategy; in the fourth column dead cell discrimination strategy by fluorescence in the PB450 channel; in the fifth column GFP associated PMN selection by fluorescence in the FITC channel and in the sixth column a scatter plot based on FITC-FSC of single-alive-PMNs.

☒ Tick this box to confirm that a figure exemplifying the gating strategy is provided in the Supplementary Information.
